# Expanding luciferase reporter systems for cell-free protein expression

**DOI:** 10.1038/s41598-022-15624-6

**Published:** 2022-07-07

**Authors:** Wakana Sato, Melanie Rasmussen, Christopher Deich, Aaron E. Engelhart, Katarzyna P. Adamala

**Affiliations:** Department of Genetics, Cell Biology and Development, University of Minnesota, Minneapolis, MN USA

**Keywords:** Biochemistry, Biological techniques, Biotechnology, Synthetic biology

## Abstract

Luciferases are often used as a sensitive, versatile reporter in cell-free transcription-translation (TXTL) systems, for research and practical applications such as engineering genetic parts, validating genetic circuits, and biosensor outputs. Currently, only two luciferases (Firefly and Renilla) are commonly used without substrate cross-talk. Here we demonstrate the expansion of the cell-free luciferase reporter system, with two orthogonal luciferase reporters: *N. nambi* luciferase (Luz) and LuxAB. These luciferases do not have cross-reactivity with the Firefly and Renilla substrates. We also demonstrate a substrate regeneration pathway for one of the new luciferases, enabling long-term time courses of protein expression monitoring in the cell-free system. Furthermore, we reduced the number of genes required in TXTL expression, by engineering a cell extract containing part of the luciferase enzymes. Our findings lead to an expanded platform with multiple orthogonal luminescence translation readouts for in vitro protein expression.

## Introduction

The cell-free transcription-translation (TXTL) is a widely used in vitro protein expression system for synthetic biology^1–3^. *E. coli*-based TXTL has been expanding its usage with intensive engineering efforts^4–8^. By coupling with reporter genes, TXTL can be used for many applications, such as viral detection^9^, metabolic modeling^10^, toxin detection^11^, biosensors^12^, and genetic circuit validation^13^. In TXTL, the most common reporter genes are luciferases and fluorescent proteins. While luciferases have a higher signal-to-noise ratio than fluorescence proteins^11^, they cannot be used to measure long-term kinetics, due to the nature of the flash reaction substrate-dependency. Thus, fluorescent proteins are preferred for measuring gene expression dynamics. Furthermore, both fluorescent proteins and luciferases are limited for their multiplexing capacity. The fluorescent proteins are limited to about four to five colors for a simultaneous measurement due to their broad emission spectra^14^. As for luciferases, although emission filters allow multiple measurements up to around six^15,16^, the number of available substrates without cross-reactivity is low. The most commonly used substrates are D-luciferin (for Firefly luciferase) and coelenterazine (for Renilla luciferase). Vargulin was recently reported for an additional no-cross-reactive substrate with *Cypridina* luciferase^17^.

Here we address two needs of the luciferase reporter systems in TXTL: expanding multiplexing capabilities, and enabling long-term kinetics measurements. We also demonstrate a TXTL extract preparation that enables the use of luciferase with minimal burden on TXTL resources.

## Results and discussions

We explored luciferase variants without substrate cross-reactivity, to construct luciferase pathways independent of substrate supplementation, and to optimize their reactions for TXTL. We focused on two luciferase systems: fungal- and bacterial-luciferases. Neither of those luciferases was previously used in TXTL, and both are capable of substrate regeneration^18–23^.

### New luciferase-substrate systems for TXTL

Here we report a successful demonstration of using fungal and bacterial luciferase-substrate systems in a bacterial TXTL. To our knowledge, neither of the reaction has been reported in TXTL.

**Figure 1:**
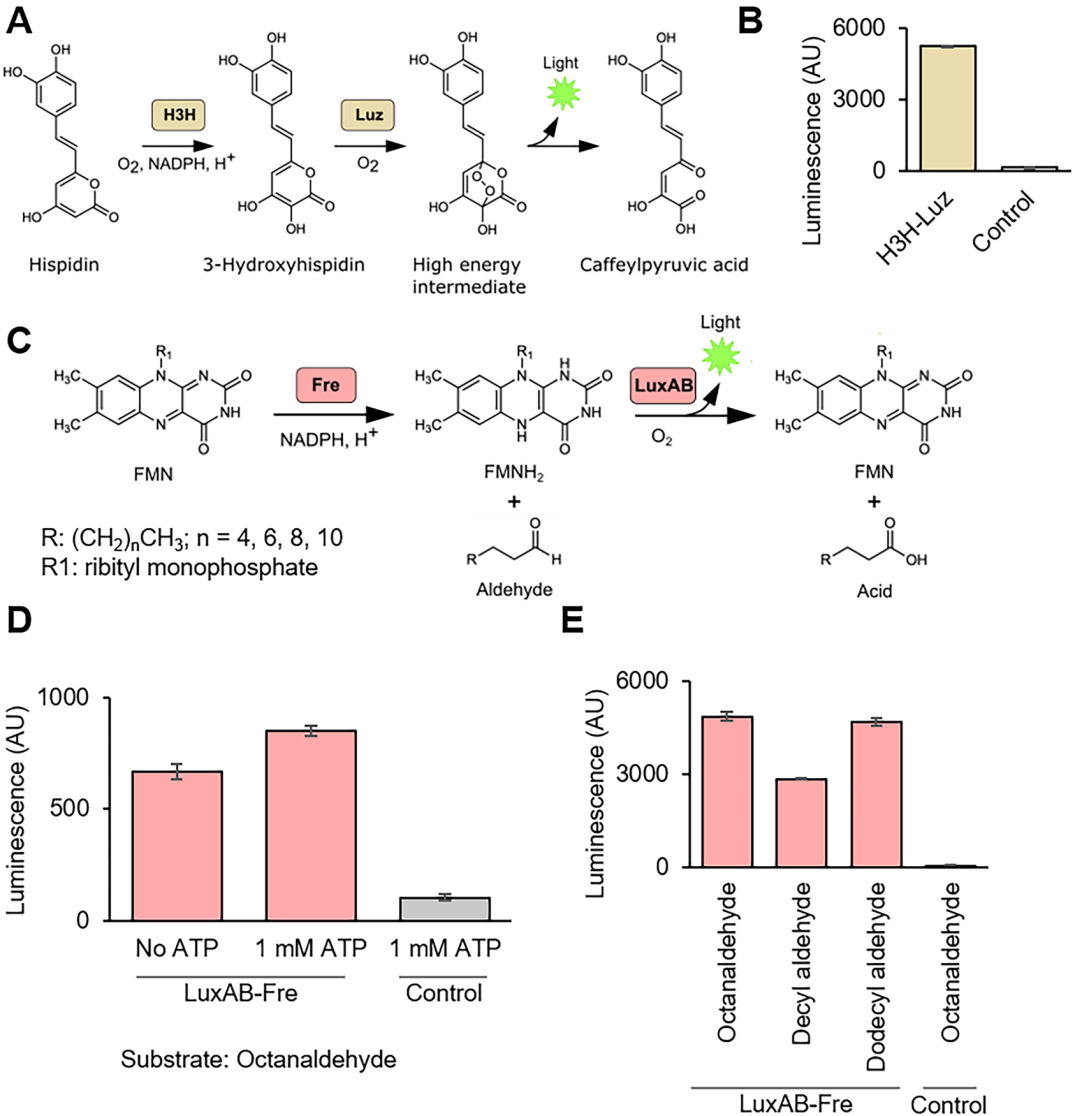
Characterization of H3H-Lux and LuxAB-Fre luciferase systems in TXTL. (**A**) Schematic of H3H-Luz luciferase reaction. Hispidin is converted to 3-hydroxyhispidin by hispidin-3-hydroxylase (H3H) and 3-hydroxyhispidin is oxidized and converted into a high energy intermediate by the luciferase (Luz). This intermediate decays into caffeylpyruvic acid with light emission. (**B**) The H3H-Luz luminescence measurement. H3H and Luz were expressed in TXTL. The luminescence was measured right after adding NADPH and hisipidin into the TXTL. (**C**) Schematic of LuxAB-Fre luciferase reaction. Oxidized flavin mononucleotide (FMN) is reduced into reduced flavin mononucleotide (FMNH_2_) by NAD(P)H-flavin reductase (Fre). The luciferase (LuxAB) converts FMNH_2_ and long-chain aldehydes into FMN and the corresponding long-chain acids with light emission. (**E**) ATP supplementation increased the light emission of LuxAB-Fre. Octanaldehyde was added as the substrate. (**D**) The LuxAB-Fre luminescence measurement with different long-chain fatty aldehydes. LuxA, LuxB and Fre were expressed in TXTL. The luminescence was measured right after adding FMN, NADPH, ATP, and substrates (octanaldehyde, decyl aldehyde, and dodecyl aldehyde.) NADPH, nicotinamide adenine dinucleotide phosphate; ATP, adenosine triphosphate; Control, reaction without enzyme expression. The graphs show means with error bars that signify SEM (n = 3).

The fungal luciferase reaction (H3H-Luz) consists of *Neonothopanus nambi* (*N. nambi*) luciferase (Luz) and *N. nambi* hispidin-3-hidroxylase (H3H). H3H converts hispidin, a commercially available chemical, to 3-hydroxyhispidin, and Luz yields light by reacting with 3-hydroxyhispidin (Fig. 1A)^18–20^. We first tested the H3H-Luz luciferase activity in TXTL. To test their activities, we individually cloned H3H and Luz genes into a vector plasmid designed for T7 RNA polymerase-coupled TXTL expression^24^, and confirmed the H3H-Luz system generated luminescence (Fig. 1B). In the reaction, we added hispidin as the substrate and NADPH as the co-factor.

The bacterial luciferase reaction (LuxAB-Fre) consists of LuxAB and NAD(P)H-flavin reductase (Fre). LuxAB is a luciferase complex that yields light by converting reduced flavin (FMNH_2_) and long-chain aldehydes into oxidized flavin mononucleotide (FMN) and the corresponding long-chain acids^21^. We combined LuxAB with Fre to reduce FMN back to FMNH_2_ (Fig. 1C)^22,25^. We cloned LuxA, LuxB, and Fre genes into the same vector plasmid used for H3H and Luz and confirmed the luminescence generation (Fig. 1D). We added octanaldehyde, FMN, and NADPH into the LuxAB-Fre luciferase reaction. Based on the mechanism known for LuxAB, ATP is not the essential compound^21,25^; however, we found ATP enhances luminescence (Fig. 1D). Thus, we supplemented ATP for the later reactions. Additionally, we tested three different long-chain fatty aldehydes with the LuxAB-Fre system and confirmed that all tested aldehydes generated luminescence (Fig. 1E). Since octanaldehyde showed strong luminescence and high solubility in the reaction, we chose octanaldehyde as the standard substrate in this report.

### Substrate specificities among different luciferase systems

**Figure 2:**
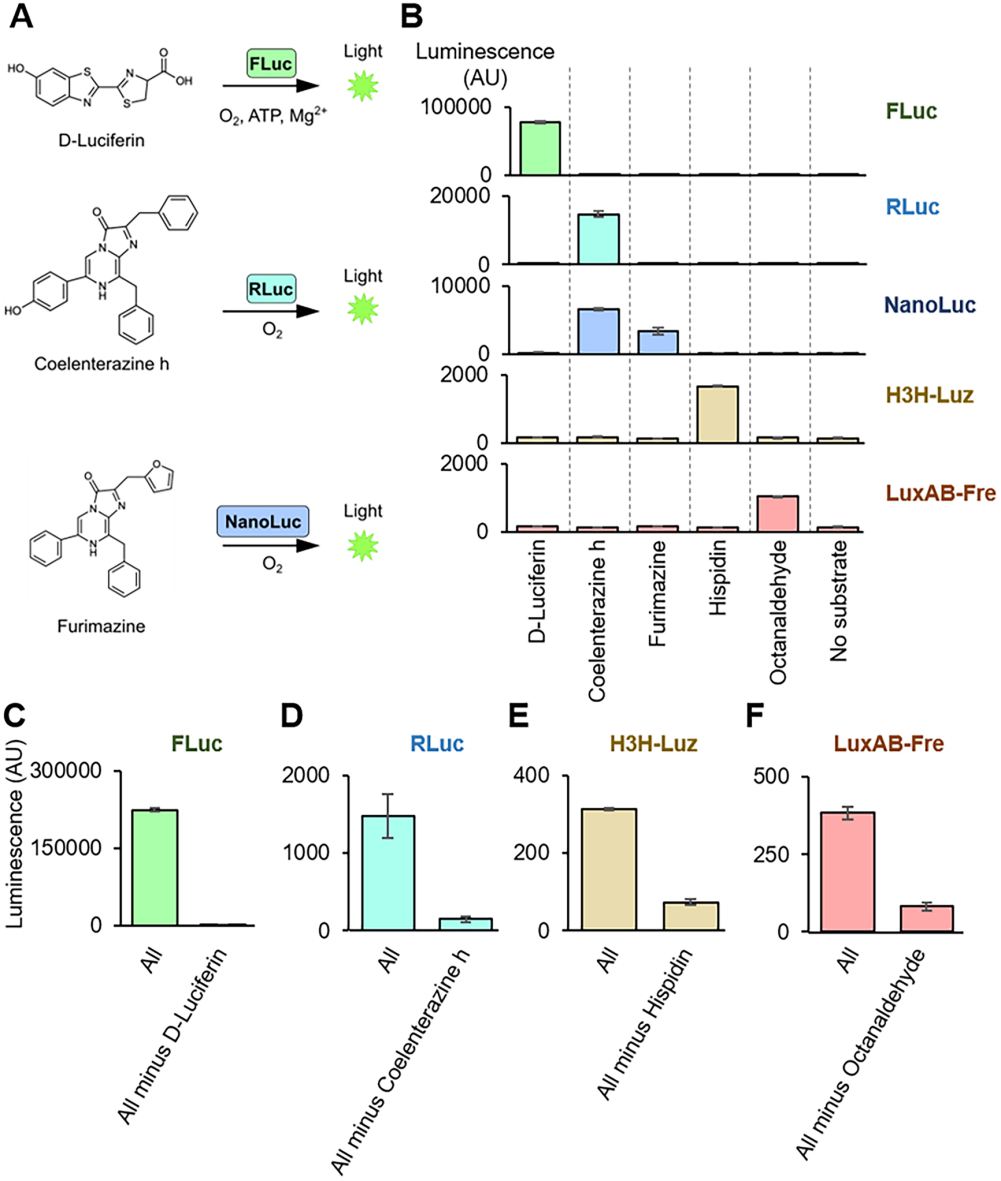
Characterization of substrate specificities. (**A**) Schematic image of firefly luciferase (FLuc), renilla luciferase (RLuc), and NanoLuc luciferase (NanoLuc) reactions. FLuc oxidizes D-luciferin with ATP and Mg^+^ to produce light. RLuc and NanoLuc oxidize coelenterazine h and furimazine, respectively, with ATP to produce light. (**B**) Luminescence measurement for substrate specificity assay for 5 luciferases. The luciferases (FLuc, RLuc, NanoLuc, H3H-Luz, and LuxAB-Fre) were expressed in TXTL. Then, the individual substrates (D-luciferin, coelenterazine h, furimazine, hispidin, and octanaldehyde) with corresponding co-factors were added to the reaction and measured its light emission without emission filters. Substrate concentrations were 10 μM, except 1 mM for octanaldehyde. (**C**–**F**) The substrate multiplexing assay. The substrate mixtures were prepared as “All” (D-luciferin, Coelenterazine h, hispidin, octanaldehyde, Mg^+^, ATP, NADPH, FMN) or “All minus one” that contains all except one that a substrate is supposed to react with a tested luciferase. The assay was performed by mixing substrates with TXTL expressing (**C**) FLuc, (**D**) RLuc, (**E**) H3H-Luz, or (**F**) LuxAB-Fre, and the luminescence was measured without emission filters. ATP, adenosine triphosphate. The graphs show means with error bars that signify SEM (n = 3).

Next, we examined the substrate specificities of H3H-Luz and LuxAB-Fre by comparing the widely used luciferase-substrate pairs: Firefly luciferase (FLuc)-D-luciferin, Renilla luciferase (RLuc)-coelenterazine h, and Nano luciferase (NanoLuc)-furimazine **(**Fig. 2A). First, we tested those substrates individually. Except for NanoLuc, all the luciferases showed significantly stronger luminescence with the substrate supposed to react than others (Fig. 2B). Then, we prepared a mixture of all four substrates (All mixture: D-luciferin, coelenterazine h, hispidin, and octanaldehyde). We tested each luciferase to see differences in the luminescence with “All mixture” or “All minus one,” which is without a suitable substrate. We omitted the NanoLuc-furimazine pair from this experiment because NanoLuc reacted with both coelenterazine h and furimazine (Fig. 2B). FLuc, RLuc, H3H-Luz, and LuxAB-Fre showed expected luminescence patterns; only the “All mixture” luminesced and the “All minus one” did not (Figs. 2C–F, S1). FLuc showed slight luminescence with the “All minus one” because of the reactivity with coelenterazine h (Fig. S2). Because the signal-to-noise ratio was significantly different, we considered this the success of differentiation. We also confirmed the enzyme expressions in the reactions by Western blot analysis (Fig. S3). The LuxAB-Fre reaction is not shown in Fig. S1, because His-tagged Fre did not show up on the membrane (LuxA and LuxB were not attached to His-tags). His-tagged Fre was detected on a membrane when expressed by itself (Fig. S4). We think the absence of the Fre band in the LuxAB-Fre reaction was because the resource competition of expressing three genes reduced each enzyme expression, resulting in invisible bands on the blot membrane. Since we detected the luminescence, we consider the enzymes were still expressed enough to generate measurable luminescence.

### Substrate regeneration with LuxABCDE-Fre system

**Figure 3:**
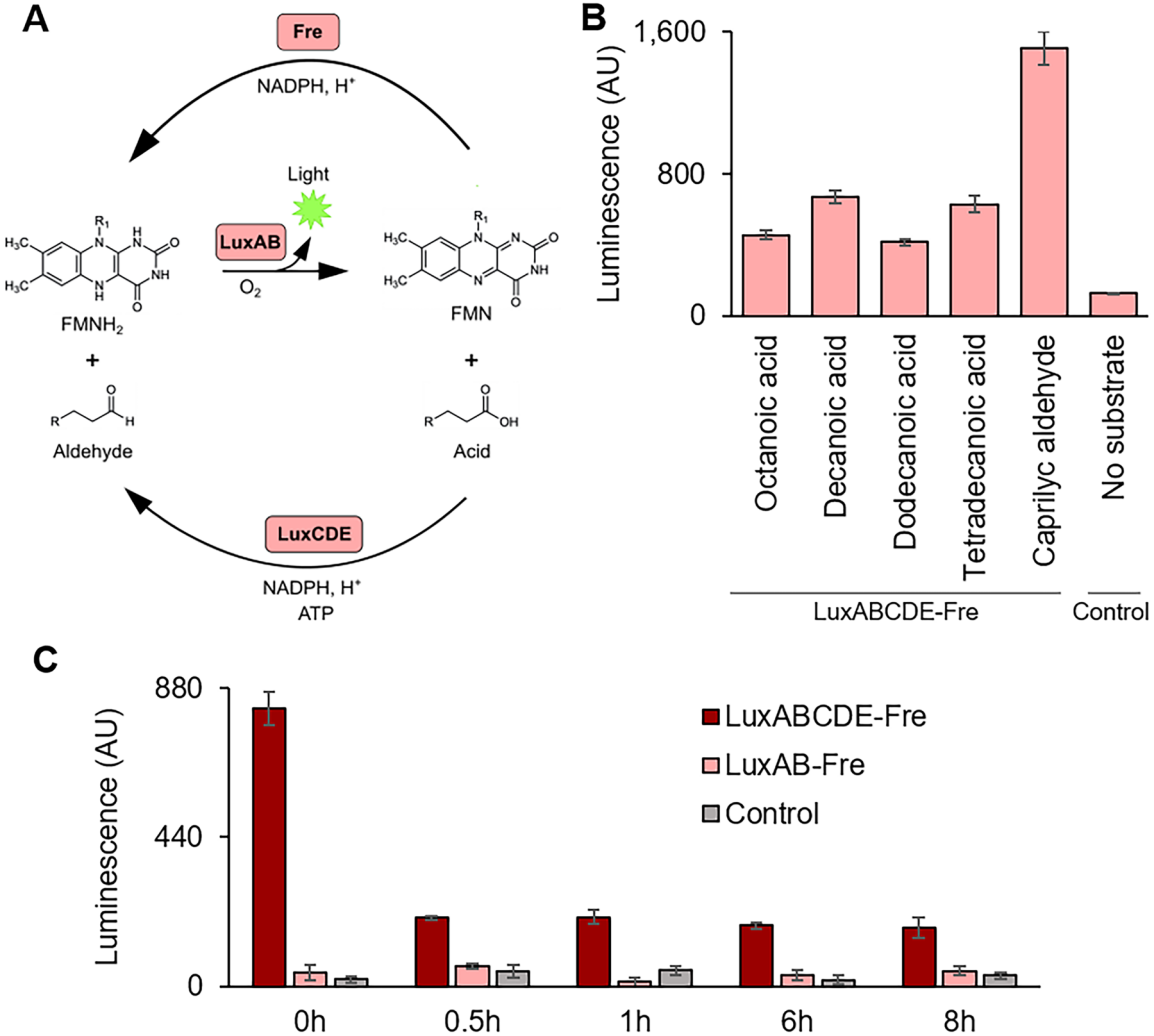
Substrate regeneration system with LuxABCDE-Fre. (**A**) The schematic of the LuxABCDE-Fre substrate regeneration system. LuxAB generates light with reduced flavin mononucleotide (FMNH_2_) and long-chain aldehyde; those substrates are converted into oxidized flavin mononucleotide (FMN) and corresponding long-chain acid. NAD(P)H-flavin reductase (Fre) reduces FMN back to FMNH_2_, and LuxCDE reduces the acid back to the corresponding aldehyde. (**B**) Luminescence measurement with the LuxABCDE-Fre system. LuxAB-Fre and LuxCDE were expressed in TXTL and mixed with 1 mM long-chain fatty acids (octanoic acid, decanoic acid, dodecanoic acid, or tetradecanoic acid) or caprylic aldehyde, followed by luminescence measurement. The reaction also contained FMN, NADPH, and ATP. Control represents a reaction using TXTL without enzyme expression. (**C**) Luminescence kinetics measurement with decanoic acid. LuxAB-Fre and LuxCDE were expressed in TXTL. For LuxABCDE-Fre reaction, the TXTL expressing LuxAB-Fre and LuxCDE were mixed with 1 mM decanoic acid (time = 0). For LuxAB-Fre reaction, the TXTL expressing LuxAB-Fre was used. For Control reaction, TXTL without enzyme expression was used. The reaction also contained FMN, NADPH, and ATP. The luminescence was measured after 0.5, 1, 6, and 8 h. The graphs show means with error bars that signify SEM (n = 3).

We propose that the LuxAB-Fre system be used for continuous luminescence reaction. LuxCDE, a protein complex encoded in another part of the LuxABCDE operon, reduces long-chain fatty acids into corresponding long-chain fatty aldehydes^21^. After the luminescence, Fre and LuxCDE can convert the FMN and long-chain fatty acids back to the LuxAB substrates. Thus, LuxABCDE-Fre can self-replenish its substrates consistently (Fig. 3A). First, we tested the luminescence production from long-chain fatty acids. This reaction requires LuxCDE to reduce long-chain fatty acids to the corresponding aldehydes. All the long-chain fatty acids we tried (octanoic acid, decanoic acid, dodecanoic acid, and tetradecanoic acid) generated luminescence (Fig. 3B). The reaction with octanaldehyde was a positive control to ensure the LuxAB-Fre working (Fig. 3B). Next, we tested whether the LuxABCDE-Fre system can regenerate the substrate to give continuous luminescence. We added the substrate (decanoic acid or octanaldehyde) into the TXTL that had expressed LuxAB-Fre and LuxCDE to incubate at 25 °C. The luminescence was measured at 0, 0.5, 1, 6, and 8 h. Only the reaction containing all the LuxABCDE-Fre enzymes retained the luminescence over 8 h (Figs. 3C, S5).

The current limitation of the LuxABCDE-Fre substrate regeneration is that we need separately express LuxAB-Fre and LuxCDE in two TXTL reactions. We tried expressing LuxABCDE-Fre in a single TXTL reaction; however, we did not detect luminescence. For the practical applications using the entire pathway, further optimization of TXTL conditions is required. We believe this demonstrated substrate regenerative pathway is still useful, particularly once we achieve the control of larger, multiple gene networks in TXTL.

We tried to reconstitute another substrate regeneration pathway from the fungal system. After Luz produces light and caffeylpyruvic acid, CPH, Hisps, and NPGA convert caffeylpyruvic acid back to hispidin (Fig. S6)^18–20^. However, we could not reproduce those reactions in TXTL, probably because we could not identify co-factors required for the reaction, or *E. coli* TXTL might not be suitable for post-translational modifications required for some, still unidentified, eukaryotic enzymes. For further detail of troubleshooting for this project, see the supplemental information section, “Efforts to engineer the fungal luciferase substrate regeneration pathway”.

### TXTL with reduced metabolic load on host cells

**Figure 4:**
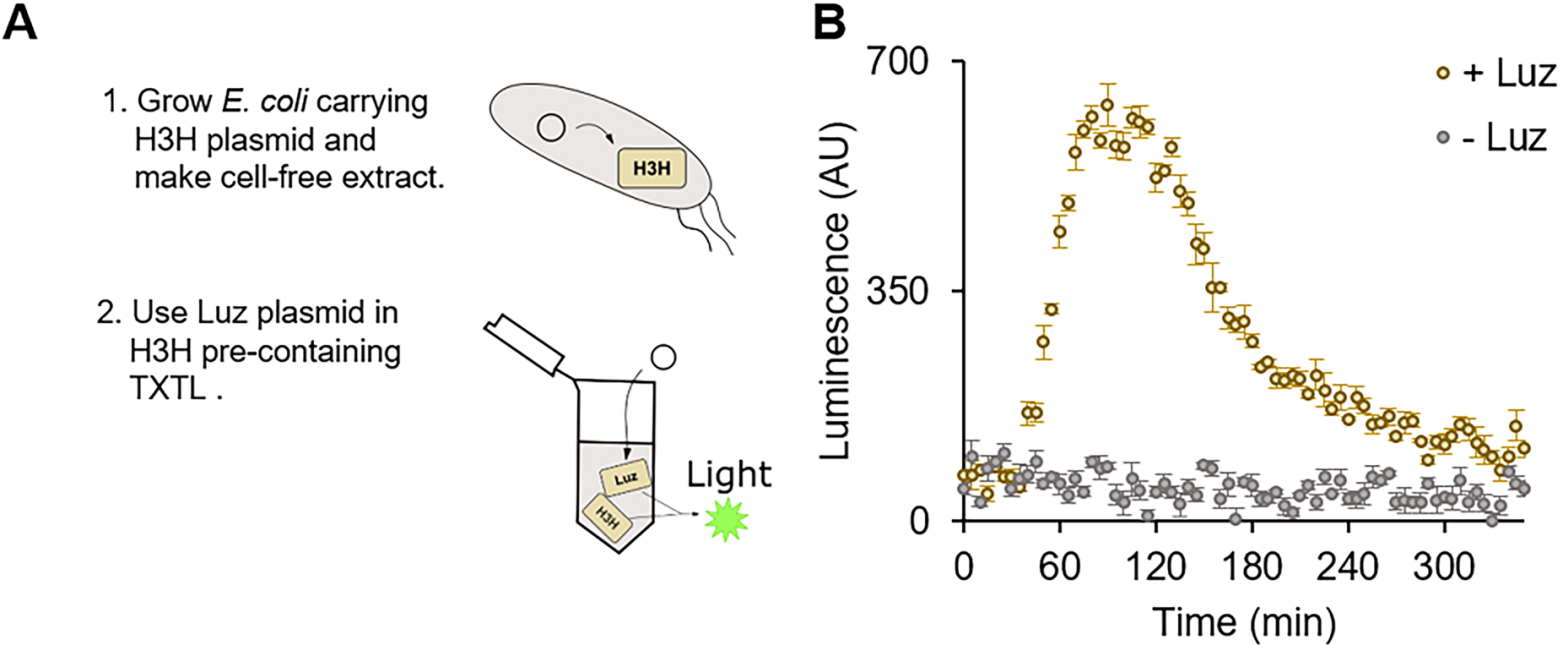
H3H-Luz tested with metabolic burden-reduced TXTL. (**A**) The schematic of how H3H-carrying TXTL works. The plasmid coding H3H gene under the sigma 70 promoter is transformed into *E. coli* Rosseta 2 strain. The cell-free extract is made with that strain; thus, the extract contains H3H. Once Luz is expressed in the TXTL, Luz produces luminescence by coordinating with H3H. (**B**) Luminescence measurement in the H3H pre-containing TXTL. Luz plasmids were incubated with hispidin at 30 °C in TXTL. The Luz plasmid containing reaction (yellow dots) generated light during the TXTL reaction, while the reaction without Luz plasmid did not (black dots.) The graphs show means with error bars that signify SEM (n = 3).

Since TXTL is a self-contained biochemical reaction, fewer genes are preferred to avoid resource competitions, which often results in poor protein expression efficiency. The H3H-Luz system, requires two genes for the reporter gene assay, may add extra metabolic burdens to TXTL compared to other single gene luciferases, such as FLuc and RLuc. To reduce the number of genes required for the reporter assay, we prepared a cell-free extract containing H3H in advance; we only needed to express Luz in TXTL for luminescence. We used *E. coli* carrying H3H plasmid in the cell-free extract preparation (Fig. 4A). This plasmid encodes the H3H gene under the *E. coli* constitutive promoter, sigma 70 promoter. We confirmed that H3H pre-expressed TXTL luminesced as Luz expressed in the presence of hispidin, while the minus Luz reaction did not (Figs. 4B, S7, S8).

We also tried this metabolic burden reduced TXTL with the LuxAB-Fre system; however, we did not make the system work with our settings. We could not detect any luminescence with TXTL using an *E. coli* extract containing all the enzymes except LuxA or LuxB.

### The capacity for reporter gene fusions

**Figure 5:**
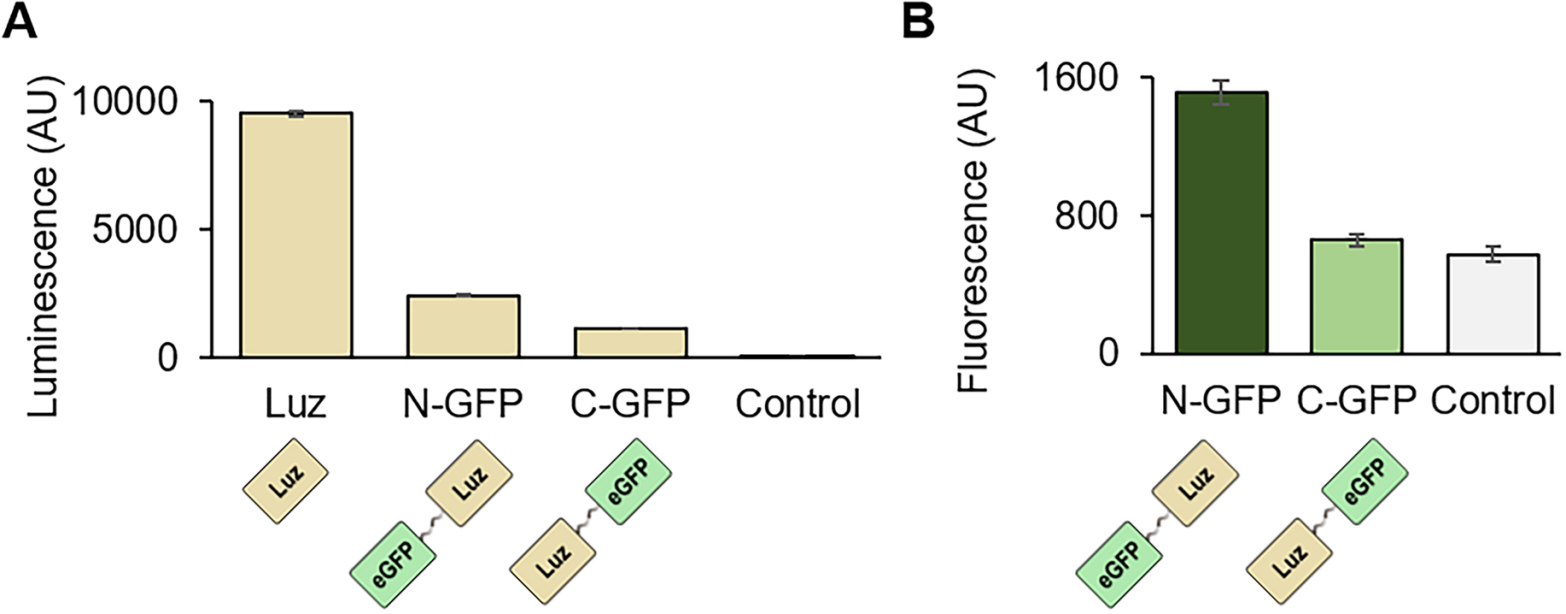
Luz’s capacity as reporter gene fusions. (**A**) Luminescence measurement of the H3H-Luz system with the eGFP fused Luz constructs. H3H and Luz were expressed in TXTL. After the expression, hispidin and NADPH were added, followed by luminescence measurement. (**B**) Fluorescence measurement of the eGFP fused Luz. Luz proteins were expressed in TXTL and the fluorescence was measured. Luz, Luz luciferase without a fusion protein; N-GFP, N-terminal eGFP fusion with Luz; C-GFP, C-terminal eGFP fusion with Luz; Control, reaction without enzyme expression. The graphs show means with error bars that signify SEM (n = 3).

Next, because reporter genes are often attached to another gene of interest, we decided to demonstrate Luz’s capacity for reporter gene fusions. We prepared two Luz-fusion constructs: His-eGFP-Luz and Luz-eGFP-His (described as N-term and C-term) 2 × GS-linkers (GGGGS) were inserted between the eGFP and Luz genes. We chose eGFP as another part of a fusion protein because of the easiness of its expression measurement, and chose GS-linker because of the most used peptide linkers. Both N-term and C-term Luz fusion constructs luminescent, although the signal intensities were lower than the Luz without eGFP fusion (Fig. 5A). The eGFP fluorescence was stronger for the N-term eGFP fusion when those fusion constructs were expressed in TXTL (Fig. 5B). 3 × GS-linkers did not improve the protein expressions compared to the 2 × GS-linkers (Figs. S9, S10). Altogether, we claim that a protein can be fused on either terminal of Luz; however, linking a protein of interest in the N-term of Luz might work better, based on the eGFP fluorescence measurement.

We tried LuxA and LuxB as reporter gene fusions; however, we could not find a construct that both fluoresced and luminesced (Fig. S11, S12, S13). We think there is still a chance that LuxA and LuxB can be used as fusion constructs with further optimizations of the combination of linkers and fusion partners.

### Optimization of the HiBiT-LgBiT system for TXTL

**Figure 6:**
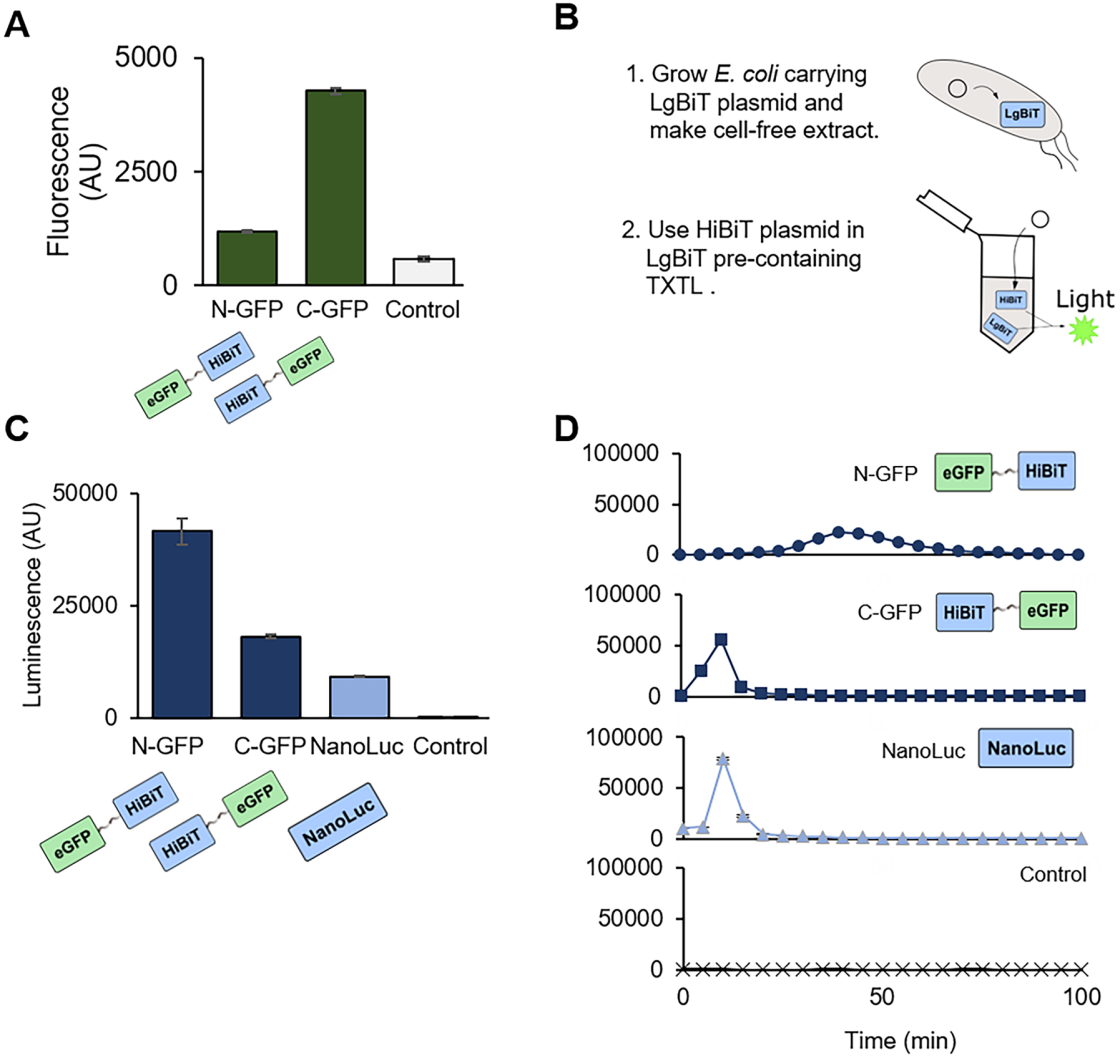
Demonstration of the use of HiBiT reporter system in TXTL. (**A**) eGFP fluorescence measurement of the HiBiT-GFP fusion proteins. Fusion proteins were expressed in TXTL at 30 °C for 8 h, followed by the fluorescence measurement. (**B**) The schematic of how LgBiT-carrying *E. coli* cell-free extract works. The plasmid coding LgBiT gene under the sigma 70 promoter is transformed into *E. coli* Rosetta 2 strain. The cell-free extract is made with that strain; thus, the extract contains LgBiT. Once HiBiT is expressed in TXTL, HiBiT produces luminescence by reconstituting a full luciferase with LgBiT. (**C**) End-point luminescence assay with the fusion HiBiTs. The fusion HiBiTs were expressed in the LgBiT-containing TXTL at 30 °C for 8 h. After the expression, 1 µM Furimazine was added, followed by luminescence measurement. (**D**) Luminescence kinetics measurement with the LgBiT-containing TXTL. HiBiT plasmids and Furimazine were added at the TXTL reaction set up and incubated at 30 °C. The luminescence was measured every 5 min during the TXTL reaction. N-GFP, N-terminal eGFP fusion with HiBiT; C-GFP, C-terminal eGFP fusion with HiBiT; Control, reaction without enzyme expression. The graphs show means with error bars that signify SEM (n = 3).

We demonstrated another metabolic burden-reduced TXTL with NanoLuc. NanoLuc can be split into two parts: LgBiT (18 kDa subunit derived from N-term NanoLuc) and HiBiT (1.3 kDa peptide, 11 amino acids, derived from C-term NanoLuc)^26,27^. First, we made HiBiT constructs linking with eGFP on either end of HiBiT. Those constructs successfully fluoresced, and the signal was stronger for eGFP on the C-term of HiBiT (Figs. 6A, S14). To make a cell-free extract, we used *E. coli* carrying a plasmid of the LgBiT gene, a bigger fragment of NanoLuc. This LgBiT-carrying TXTL only requires 11 amino acids (HiBiT) expression for luminescence generation (Fig. 6B). With end-point measurement, we confirmed the N-term and C-term HiBiT fusion constructs generated brighter signals than NanoLuc in the LgBiT carrying TXTL, and the N-term fusion produced the brightest luminescence (Fig. 6C). Next, we measured the kinetics of luminescence during the HiBiT expressing TXTL reactions. HiBiT with C-term eGFP fusion generated luminescence earlier (max at 10 min) than with N-term eGFP fusion (max at 40 min) (Figs. 6D, S15, S16). This timing differences are probably because the N-term eGFP fusion construct cannot complete NanoLuc formation and luminesce until the whole GFP-NanoLuc is expressed. In contrast, the C-term fusion expresses HiBiT first.

### Summary and perspectives

In this work, we established the use of two new luciferase systems as protein expression reporters in cell-free translation systems, and we demonstrated a technique that minimizes the metabolic burden on TXTL while using the split luciferase assay. The work presented in this paper expands the toolbox of luminescent protein reporters for cell-free applications, building a more complete and versatile platform for a variety of cell-free and synthetic cell applications.

## Materials and methods

DNA oligonucleotides were purchased from Integrated DNA Technologies (IDT). Thermal cyclers used for sample incubation were Bio-Rad T100 thermo cyclers running software version 1.201. A plasmid for fungal luciferase pathway, P307-FBP_6, was a gift from the Daniel Voytas lab at the University of Minnesota^20^. Cloning vector plasmids, pCI-T7Max-UTR1-CTerminus8xHis-T500 and pCI-T7Max-UTR1-NTerminus8xHis-T500, were obtained from our lab stock^24^. Plasmids for other luciferases, pGreen_dualluc_3'UTR_sensor, pGEN-luxCDABE, pUAS-NanoLuc, and pBad-LgBiT-PhoCl1-SmBiT-MBP, were purchased from Addgene^28–30^.

### TXTL reactions

This protocol was adapted from Noireaux^4^ and Jewett^5^ protocols. The Rosetta 2 (Novagen, 71,400) cell extract preparation was followed by the method described previously^31^ with one modification. A 750 ml 2xYPTG was grown at 30 °C instead of 37 °C. For H3H or LgBiT containing TXTL, Rosetta 2-derived strains carrying pLumi-H3H or pLumi-LgBiT were used for cell extract preparation. The electrocometent cells were prepared from Rosetta 2 *E. coli*, and the plasmid, pLumi-H3H or pLumi-LgBiT, was transformed. The successful transformant was selected through ampicillin resistance.

Cell‐free transcription‐translation (TXTL) reactions were composed of the following: 12 mM Magnesium glutamate; 140 mM potassium glutamate; 1 mM DTT; 1.5 μM T7 RNA polymerase; 0.4 U/µl Murine RNase Inhibitor (NEB, M0314S); 1 × cell-free prep; 1 × energy mix; and 1 × amino acid mix. The plasmid concentrations were 15 nM. Unless otherwise specified, the TXTL reactions were incubated at 30 °C for 8 h, followed by 4 °C hold.

10 × Energy mix composition was the following: 500 mM HEPES, pH 8; 15 mM ATP; 15 mM GTP; 9 mM CTP; 9 mM UTP; 2 mg/mL E. coli tRNA; 0.68 mM Folinic Acid; 3.3 mM NAD; 2.6 mM Coenzyme-A; 15 mM Spermidine; 40 mM Sodium Oxalate; 7.5 mM cAMP; 300 mM 3-PGA.

10 × amino acid mix was prepared by mixing 20 mM of the following amino acids: alanine, arginine, asparagine, aspartic acid, cysteine, glutamic acid, glutamine, glycine, histidine, isoleucine, leucine, lysine, methionine, phenylalanine, proline, serine, threonine, tryptophan, tyrosine, and valine. Those amino acids were dissolved in pH 6.5, 400 mM potassium hydroxide solution.

### Western blot analysis

The Western blot was performed with a method described previously^24^. The samples were fractionated on a 37.5:1 acrylamide:bis-acrylamide SDS-page gel at 100 V in 800 ml 1 × SDS running buffer (25 mM Tris, 192 mM Glycine, 3.5 mM SDS). The gel percentage and fractionation time varied and are indicated on each figure. The blots were imaged by ChemiDoc MP Imaging System with Image Lab Software (BIORAD), with the image application Blots, Chemi hi sensitivity reagent and Colorimetric. The chemiluminescent blot image and colorimetric image of the same blot were combined using the software merging function.

### Luciferase assays

#### Substrate preparation

The chemicals used in the luciferase assay were as follows: FMN-Na (Alfa Aesar, J66949.09), NADPH (Cayman Chemical Company, 9000743), ATP (Larova GmbH, ATP_100ML), D-luciferin (Cayman Chemical Company, 25836), coelenterazine (Cayman Chemical Company, 16123), coelenterazine H (Promega, S2011), frimazine (Aoblous, AOB36539), hispidin (Cayman Chemical Company, 10012605), octanaldehyde (Fisher Scientific, O004425ML), decyl aldehyde (Fisher Scientific, AC154971000), dodecyl aldehyde (fisher scientific), octanoic acid (Fisher Scientific, O002725ML), decanoic acid (Fisher Scientific, AC167271000), dodecanoic acid (Fisher Scientific, S25377), tetradecanoic acid (Fisher Scientific, AAA1206730). D-luciferin, furimazine, and hispidin were dissolved in DMSO as 10 mM stocks. Coelenterazine and Coelenterazine H were dissolved in ethanol as 10 mM stocks. Long-chain fatty aldehydes and acids were dissolved in ethanol as 500 mM stocks. Dodecyl aldehyde was not soluble in 100% ethanol at the concentration of 500 mM; we used the suspension with vortexing each time.

#### Luminescence measurement setting

The luminescence measurements were performed with SpectraMax Gemini EM Microplate Reader or SpectraMax Gemini Microplate Reader. The readings were performed by measuring the luminescence of all the wavelengths with readings “6” and photomultiplier tube (PMT) setting “medium”. 15 µl of samples were transferred to a 384 well white flat bottom assay plate (Corning®, 3705) and measured. For the kinetics measurements, the plate was sealed tightly to avoid evaporation.

#### End-point luciferase assay

Enzymes were expressed in 20 μl TXTL with each plasmids’ concentration of 15 nM at 30 °C for 6 h. Then, in 50 μl luciferase reactions, the 20 μl TXTL, substrates, and co-factors were mixed. The substrate concentrations were 1 mM for aldehydes (octanaldehyde, decyl aldehyde, or dodecyl aldehyde) or 10 μM for other substrates (D-luciferin, coelenterazine H, Furimazine, and Hispidin). FLuc reaction contained 5 mM MgCl_2_ and 1 mM ATP; fungal luciferase (H3H-Luz) reaction contained 1 mM NADPH; LuxAB + Fre reaction contained 100 μM FMN, 1 mM NADPH, and 1 mM ATP. For multiplexing assay, luciferase reactions were prepared with “All” the substrates or “All minus one” substrate. The substrate concentrations were 1 mM for octanaldehyde and 10 μM of other substrates (D-luciferin, coelenterazine H, Furimazine, and Hispidin). The reaction also contained 1 mM MgCl_2_, 1 mM ATP, 1 mM NADPH, and 100 μM FMN.

Immediately after mixing the reaction, luminescence was measured by a plate reader. For the control, water was added instead of the components.

#### LuxCDABE-Fre substrate regeneration assay

For the end-point measurement, TXTL 1 and TXTL 2 were prepared separately. TXTL 1 contained 15 nM LuxA, LuxB, and Fre plasmids, and TXTL 2 contained 15 nM LuxC, LuxD, and LucE plasmids. After incubating TXTL at 30 °C for 6 h, luciferase reactions were prepared with TXTL 1 and 2. The 50 μl luciferase reactions contained 20 μl TXTL 1, 20 μl TXTL 2, 100 μM FMN, 1 mM NADPH, 1 mM ATP, and 1 mM substrates (octanoic acid, decanoic acid, dodecanoic acid, 1-tetradecanoic acid, or octanaldehyde.) Immediately after mixing the luciferase reaction, the luminescence was measured by a plate reader. For the control reaction, water was added into the TXTL instead of the plasmid. The reaction components for the kinetics measurement were the same as the end-point measurement. The reading was performed every 5 min for 8 h.

#### eGFP fluorescence measurement

The fluorescence was measured at λ_ex_ 488 nm and λ_em_ 509 nm with plate reader PMT setting “medium” and 6 reads per well. All fluorescence measurements were performed on SpectraMax. For the endpoint measurement, 19 μl of TXTL reaction was transported into a 384 black bottom well plate to measure.

#### RT-qPCR

The DNA in 2 μl of TXTL reaction was degraded with 0.5 μl of TURBO DNase (2 U/μl, Invitrogen, AM2238) at 37 °C for 30 min. The TXTL reaction was quenched by addition of 15 mM EDTA and incubated at 75 °C for 10 min. The denatured proteins were pelleted through centrifugation at 3200 × g for two minutes. To prepare a 20 μl reverse transcription reaction, 2 μl of DNase-treated sample was mixed with 1 μM reverse primer (Luz: TTTGGCATTCTCGACGATTTTAC, HiBiT-eGFP fusion constructs: GATCCCGGCGGC), 10 mM DTT, 0.5 mM dNTP (Denville, CB4430-2), 5 U/μl protoscript II reverse transcriptase (NEB, M0368X), 1 × protoscript II reverse transcriptase buffer, and 0.4 U/μl Murine RNase Inhibitor (NEB, M0314S). The reverse transcription was performed at 42 °C for one hour, followed by the inactivation at 65 °C for 20 min. A 25 μl qPCR reaction was performed by mixing the following: 1 μl of the reverse transcribed DNA, 0.8 μM forward (Luz: CTGTGGAGTTGTCCTCG, HiBiT-eGFP fusion constructs: AAGTTCATCTGCACCACC) and reverse (Luz: GTGTGAGGTAATACTCGGTC, HiBiT-eGFP fusion constructs: TTGAAGTCGATGCCCTTC) primers, 1 × OneTaq Hot Start 2X Master Mix with Standard Buffer (NEB, M0484L), and 1 × Chai Green Dye (CHAI, R01200S). The qPCR was performed on CFX96 Touch Real-Time PCR Detection System (BioRad). The thermocycling program was set up as follows: one cycle of 30 s denaturation at 95 °C, 30 cycles of 15 s denaturation at 95 °C, 15 s annealing at 50 °C, 30 s extension at 68 °C, and one cycle of five minutes final extension at 68 °C. The amplification curves plotted through CFX Maestro Software to determine Cq values and averaged across three replicates of each sample were calculated separately.

## Supplementary Information

Supplementary Information.

## Data Availability

All relevant data are within the manuscript and its supplementary Information file.
