# Supplementary material for: Expanding luciferase reporter systems for cell-free protein expression
Source: Sci Rep. 2022 Jul 7;12:11489. doi: 10.1038/s41598-022-15624-6 (PMC9263134; doi:10.1038/s41598-022-15624-6)
Supplement: Supplementary file 1 — Supplementary Information. [file 41598_2022_15624_MOESM1_ESM.pdf]

Supplementary information

for manuscript

Expanding luciferase reporter systems for cell-free protein expression

by

Wakana Sato, Melanie Rasmussen, Christopher Deich, Aaron E. Engelhart, Katarzyna P. Adamala

Figure S1

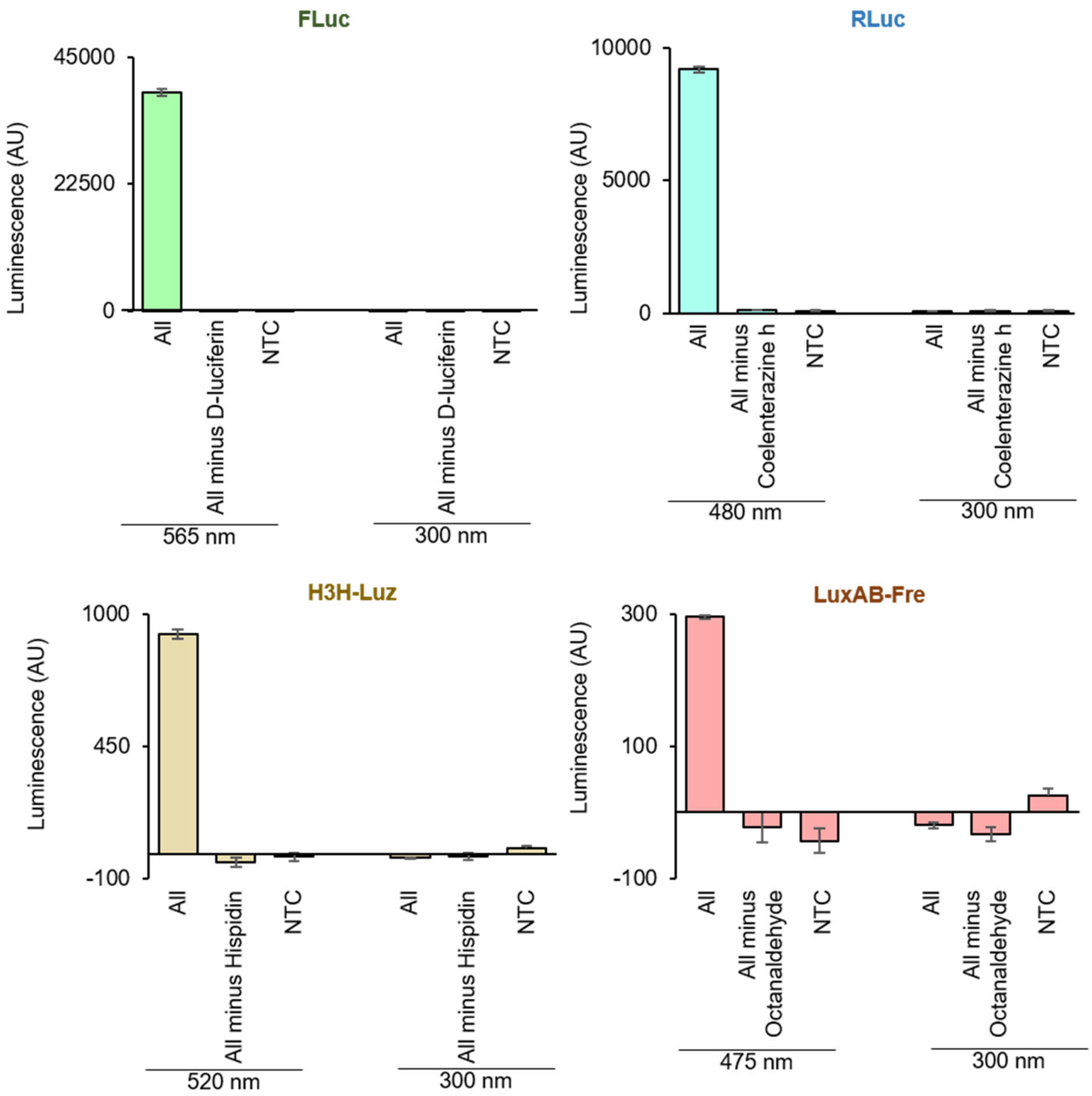

Figure S1 Luminescence measurements with emission filters.

Luminescence signals were measured with emission filters at the wavelength appropriate for each luciferase. The substrate mixtures were prepared as "All" (D-luciferin, Coelenterazine h, hispidin, octanaldehyde, Mg^{+}, ATP, NADPH, FMN) or "All minus one" that contains all except one that a substrate is supposed to react with a tested luciferase. The assay was performed by mixing substrates with TXTL expressing each luciferase. The emission filter at 300 nM was used for the negative control. The graphs show means with error bars that signify SEM (n = 3).

Figure S2

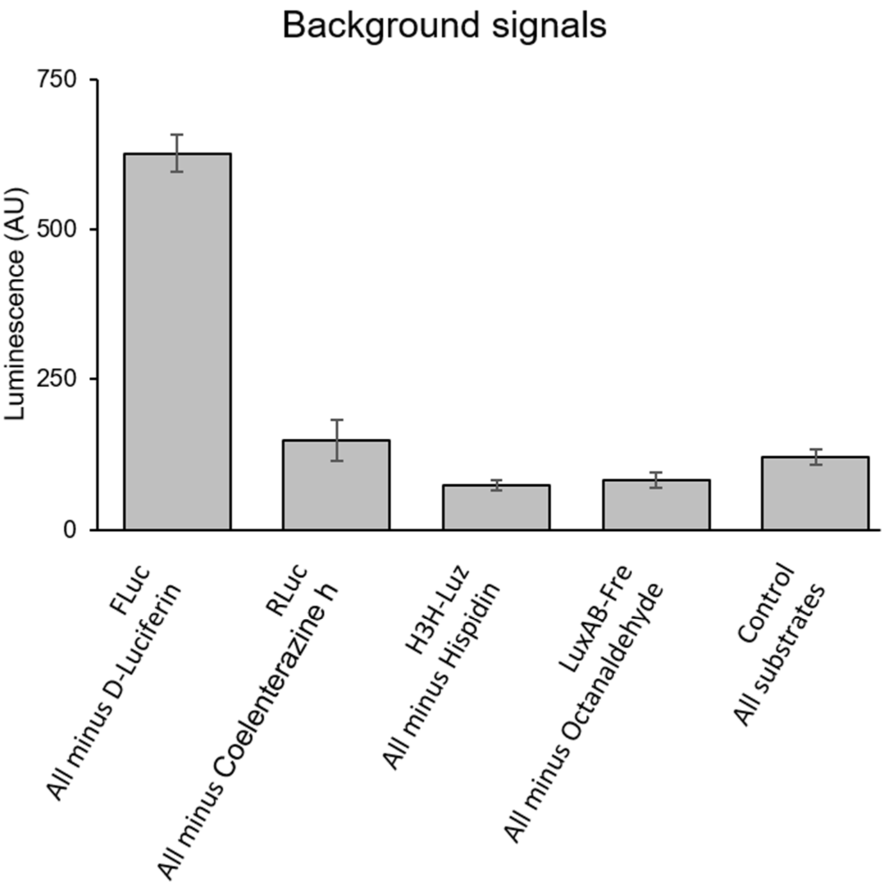

Figure S2

Background signals of the substrate specificity assay. The luciferases used in the reaction are indicated as FLuc, RLuc, H3H-Luz, or LuxAB-Fre. Control stands for reaction without enzyme expression. Substrates in the reaction were indicated as “All” (D-luciferin, coelenterazine h, hispidin, octanaldehyde) or “All minus one”, that one is the substrate supposed to react with the tested luciferase. The graphs show means with error bars that signify SEM (n = 3).

Figure S3

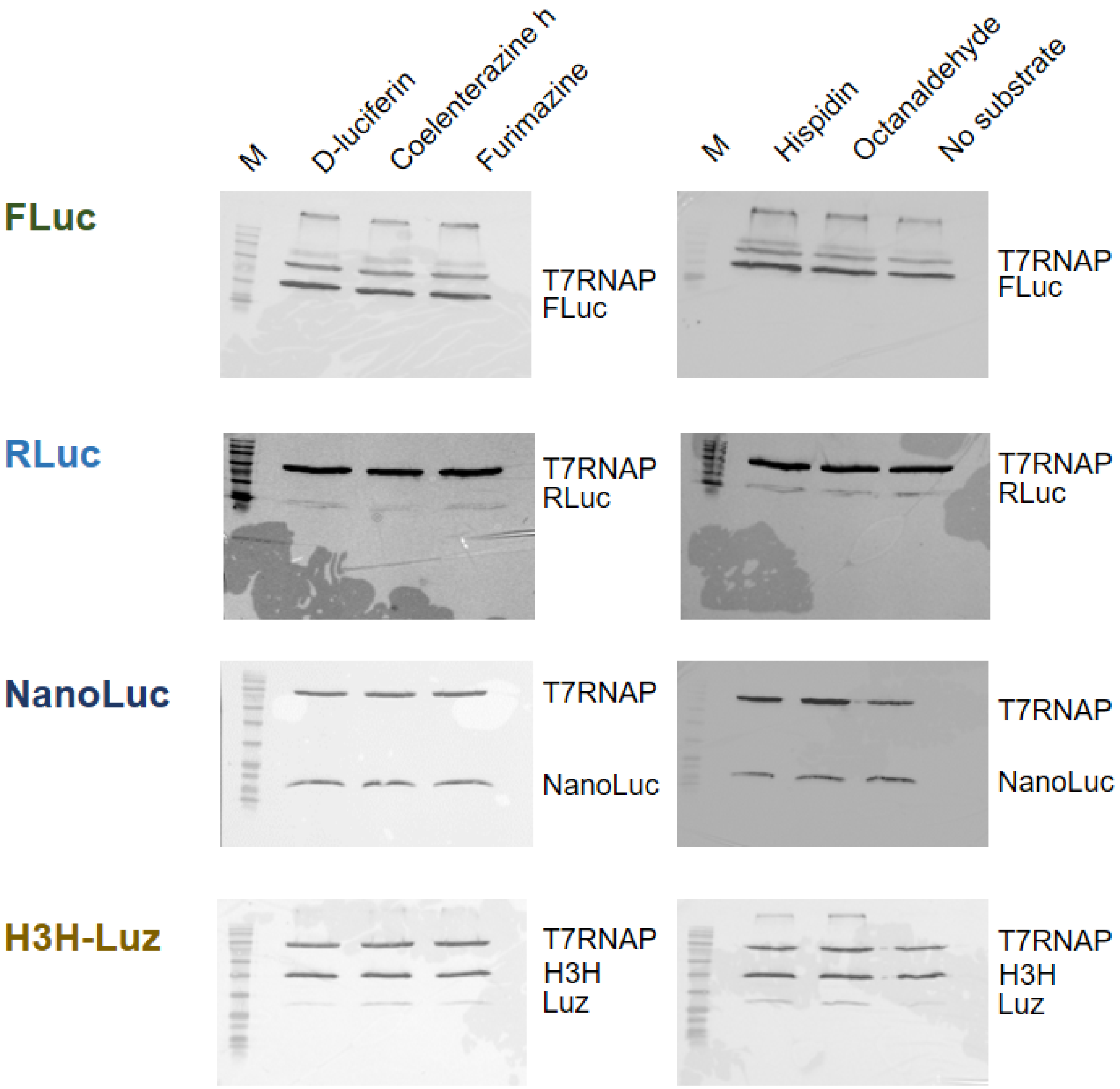

Figure S3

Western blot gels from the substrate specificity reactions. The loaded samples directly came from the reactions shown in **Fig. 2B**, indicating that the luciferase enzymes were expressed in the reactions. 15 µl of TxTL was loaded on each lane. FLuc and RLuc samples were fractionated on a 7.5% gel for 70 minutes at 100V. NanoLuc and H3H-Luz samples were fractionated on a 12% gel for 80 minutes at 100V. M, BLUEstain 2 Protein ladder, 5-245 kDa (Goldbio, P008-500); substrate names, a substrate that is contained in the reaction; T7RNAP, N-terminal His-tagged T7 RNA polymerase; FLuc, firefly luciferase with C-terminal His-tag; RLuc, Renilla luciferase with C-terminal His-tag; NanoLuc, NanoLuc luciferase with C-terminal His-Tag; H3H, hispidin-3-hydroxylase with C-terminal His-tag; Luz, fungi luciferase with C-terminal His-tag. Fig. S21 are the original blot images.

Figure S4

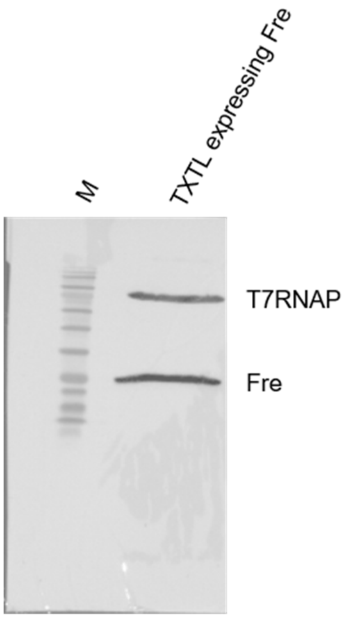

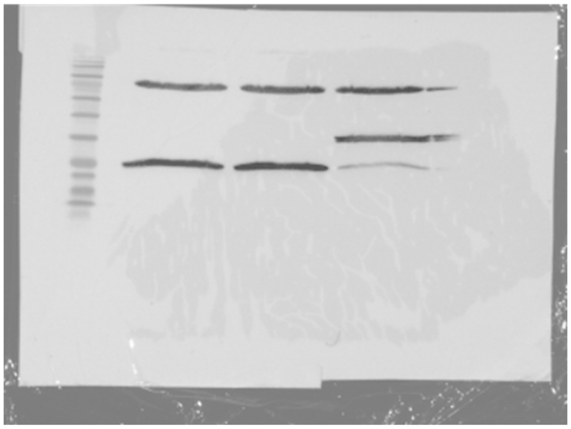

Figure S4 Western blot of Fre.
10 µl of TXTL expressing Fre was loaded on the right lane and fractionated on a 12% gel for 80 minutes at 100V. The left lane labeled with M stands for BLUEstain 2 Protein ladder, 5-245 kDa (Goldbio, P008-500). The bottom image is the original data. Samples loaded next to the Fre were TXTL reactions expressing His-tag proteins (Fre in the middle lane, LuxAB and LuxG in the left lane).

Figure S5

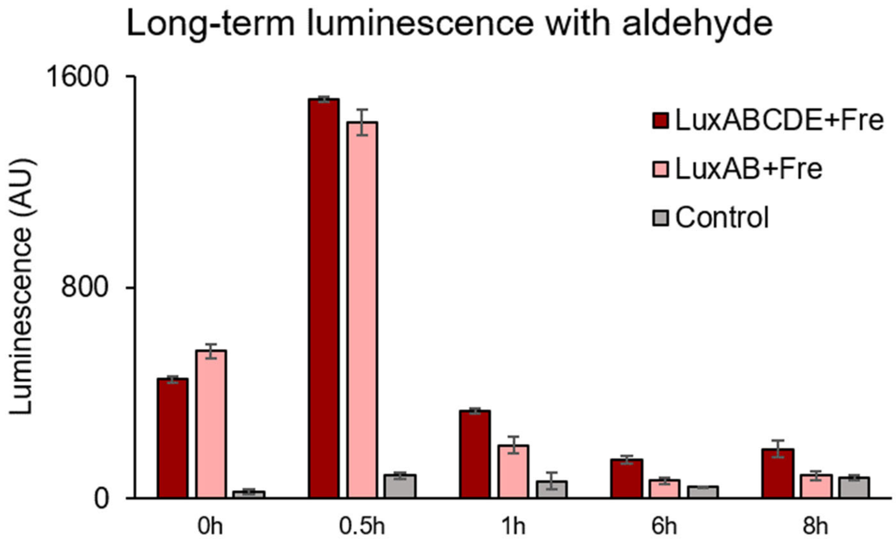

Figure S5 Luminescence kinetics measurement with octanaldehyde.
After expressing LuxABCDE+Fre or LuxAB+Fre in TXTL, 1 mM octanaldehyde was added as the substrate (time = 0). The luminescence was measured after 0.5, 1, 6, 8 hours. LuxABCDE-Fre, a reaction with TXTL expressing LuxAB-Fre and LuxCDE; LuxAB-Fre, a reaction with TXTL expressing LuxAB-Fre; Control, reaction with TXTL without enzyme expression. The graphs show means with error bars that signify SEM (n = 3).
In this experiment, the luminescence was the highest at 0.5 hours, while the starting time (t = 0) was the highest in the similar experiment using decanoic acid (**Fig. 3C**). This luminescence delay was caused probably because we held mixed reactions on ice for 10 minutes, during the recovery of the connection error between a plate reader and a computer. We think this holding on ice cooled the reactions and caused the delay reaching the maximum luminescence**.**

Figure S6

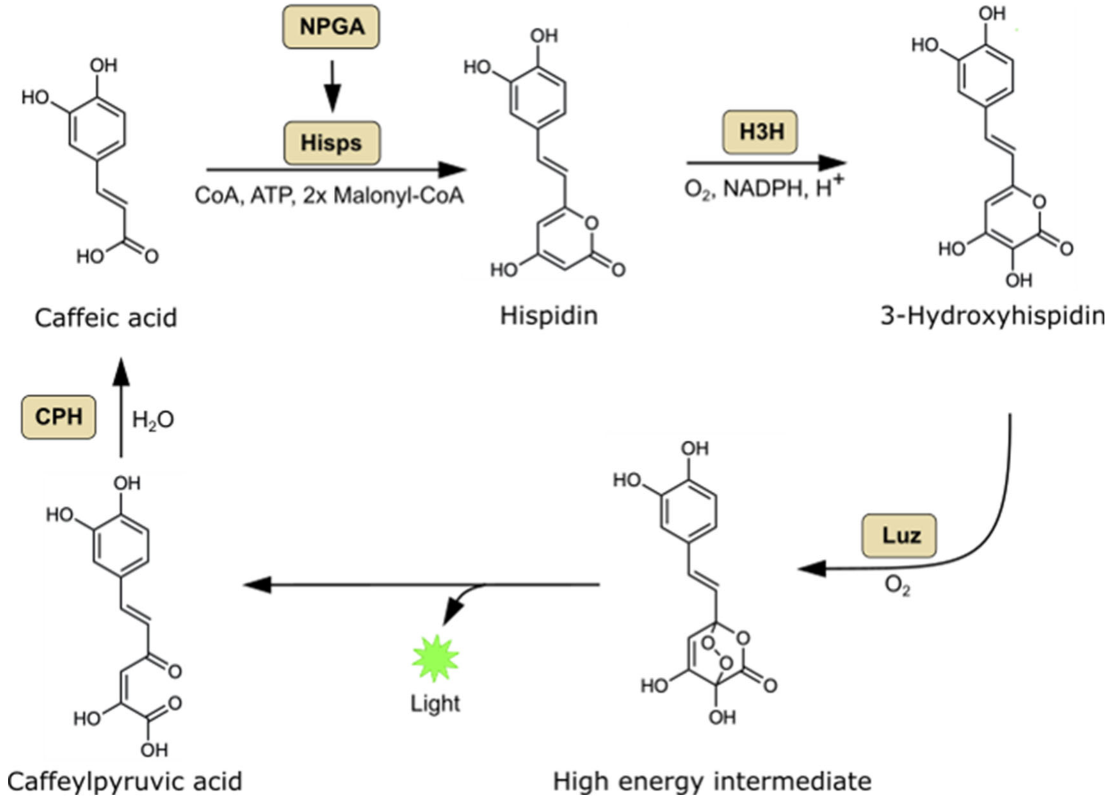

Figure S6 The proposed fungi luciferase substrate regeneration pathway.
This figure was adapted from Khakhar et al. paper[20]. In the fungi luciferase system, hispidin is converted to 3-hydroxyhispidin by *Neonothopanus nambi* (*N. nambi*) hispidin-3-hydroxylase (H3H), and then *N. nambi* luciferase (Luz) yields light by reacting with 3-hydroxyhispidin. Caffeylpyruvic acid can be recycled into caffeic acid by caffeylpyruvate hydrolase (CPH). Caffeic acid can be converted into hispidin by hispidin synthase (Hisps). Hisps needs to be post-translationally activated by 4'-phosphopantetheinyl transferase (NPGA)[18-20].

Figure S7

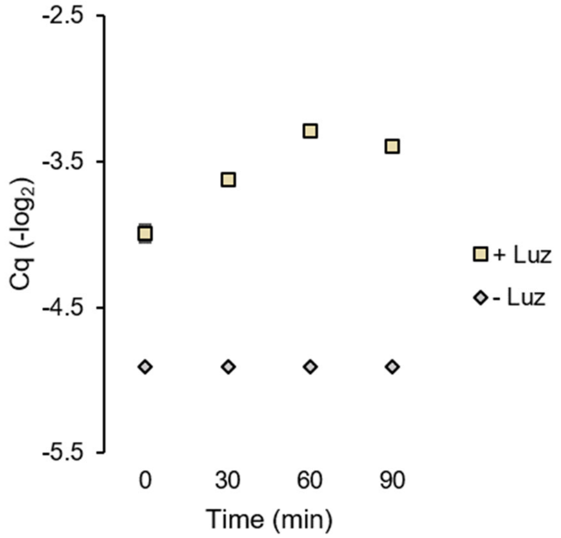

Figure S7 Luz mRNA abundance in TXTL
RT-qPCR was performed to monitor the Luz mRNA level during the **Fig. 4B** kinetic reactions. The graphs show means with error bars that signify SEM (n = 3).

Figure S8

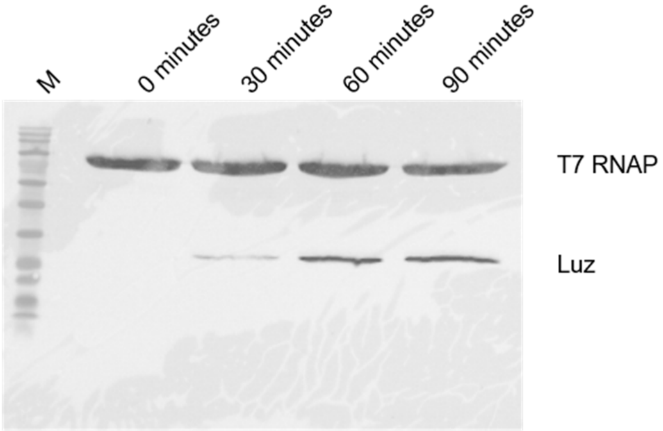

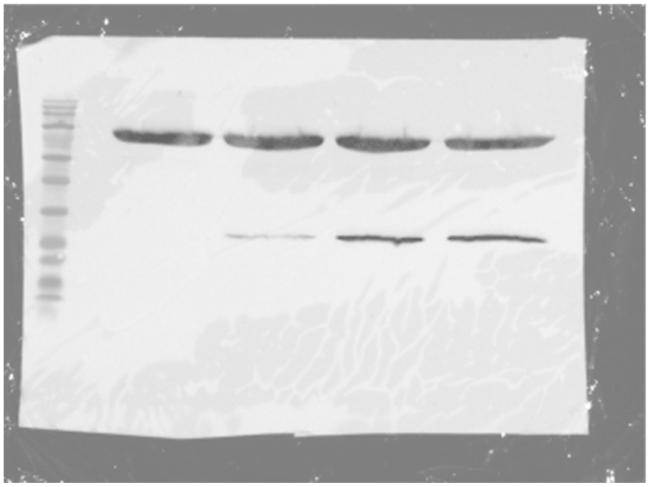

Figure S8 Luz protein abundance in TXTL.
Western blot of Lux-expressing TXTL during the **Fig. 4B** kinetic reactions**.** 15 µl of TXTL was loaded on each lane and fractionated on a 12% gel for 90 minutes at 100V. The lane labeled with M stands for BLUEstain 2 Protein ladder, 5-245 kDa (Goldbio, P008-500). The bottom image is the original data.

Figure S9

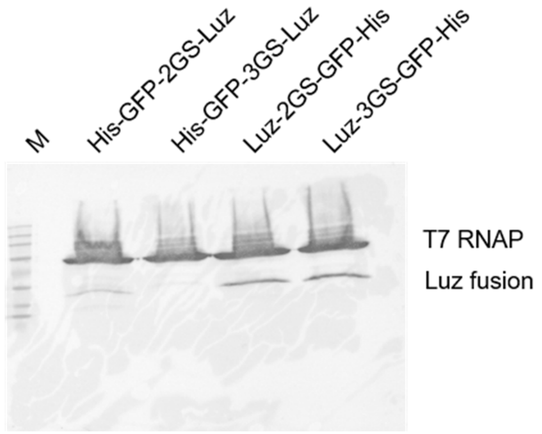

Figure S9 Luz-fusion gel image.

Western blot of Luz-GFP fusion proteins. 15 µl of TXTL was loaded on each lane and fractionated on a 7.5% gel for 60 minutes at 100V. The lane labeled with M stands for BLUEstain 2 Protein ladder, 5-245 kDa (Goldbio, P008-500). The protein names used for lane labels represent the fusion protein constructs from N-terminal to C-terminal order. His, His-Tag; GFP, enhanced green fluorescent protein; 2GS, two GS-linker sequence (GGGGS) repeats; 3GS, three GS-linker sequence repeats; T7 RNAP, T7 RNA polymerase. The bottom image is the original data.

Figure S10

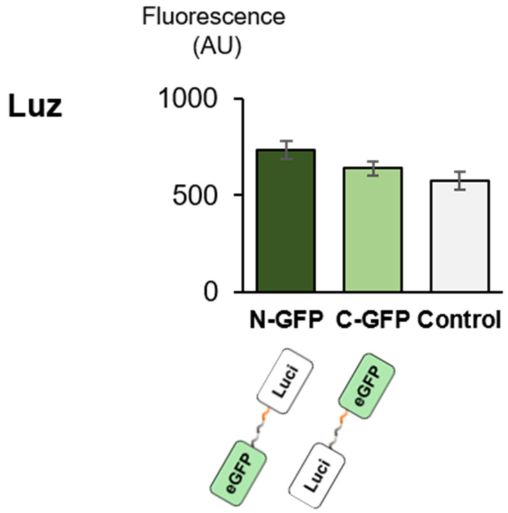

Figure S10 Fluorescence generated from eGFP-luciferase fusion proteins (Luz) with extended GS-linker.

All fusion proteins were expressed in TXTL at 30 ˚C for 8 hours, followed by fluorescence measurement. 19 µl of TXTL was used for the measurement. eGFP and luciferases are linked through 3x GS-linker (GGGGS.) Control stands for a reaction without protein expression.

Figure S11

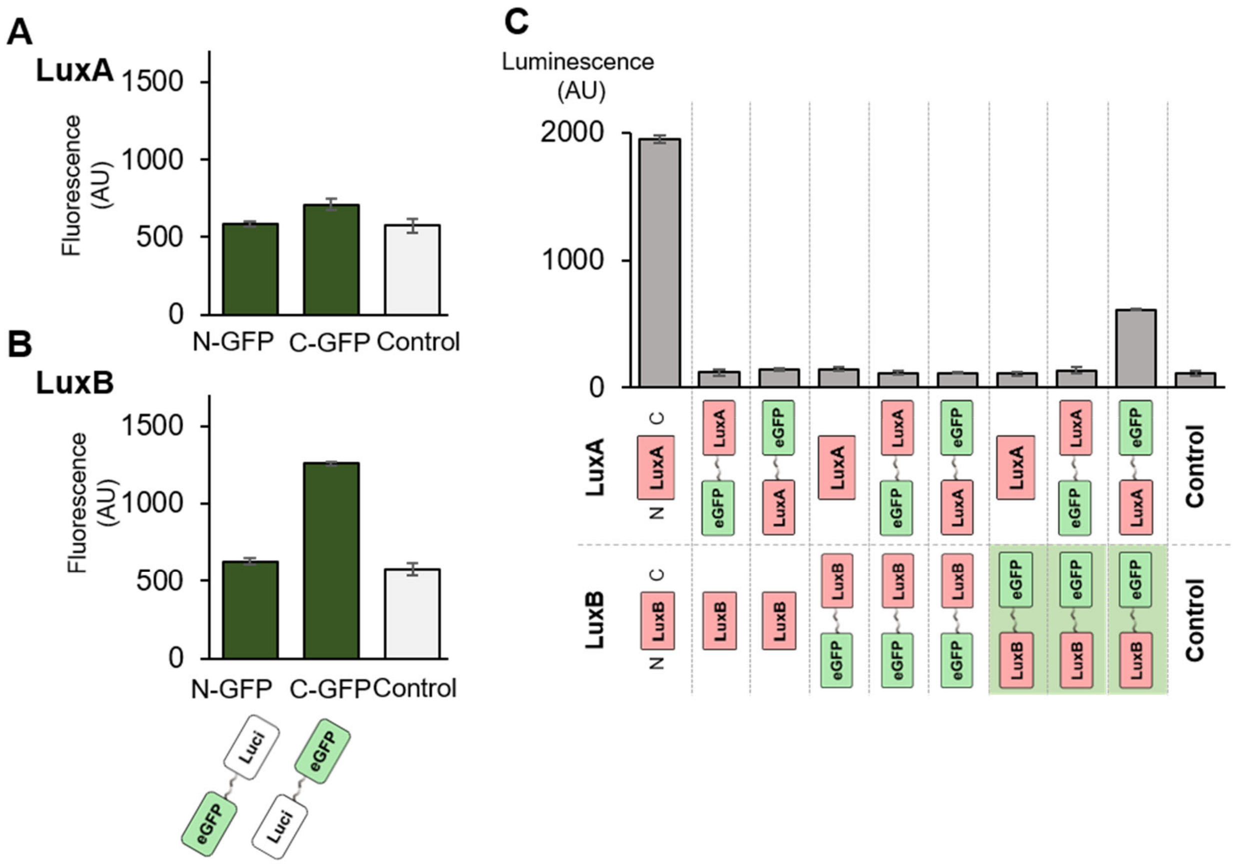

Figure S11 LuxA and LuxB capability as fusion proteins.

(A and B) eGFP fluorescence measurement in TXTL. Fusion LuxA and LuxB were expressed in TXTL at 30°C for 8 hours, followed by the fluorescence measurement. (C) Luminescence measurement with all the combinations of LuxA and LuxB fusion constructs. LuxA and LuxB were expressed in TXTL, and then 1 mM Octanaldehyde was added, followed by luminescence measurement. Green shading behind the construct images indicates that the constructs fluorescence when expressed in TXTL. N-GFP, N-terminal eGFP fusion luciferase; C-GFP, C-terminal eGFP fusion luciferase; Control, reaction without enzyme expression.

Figure S12

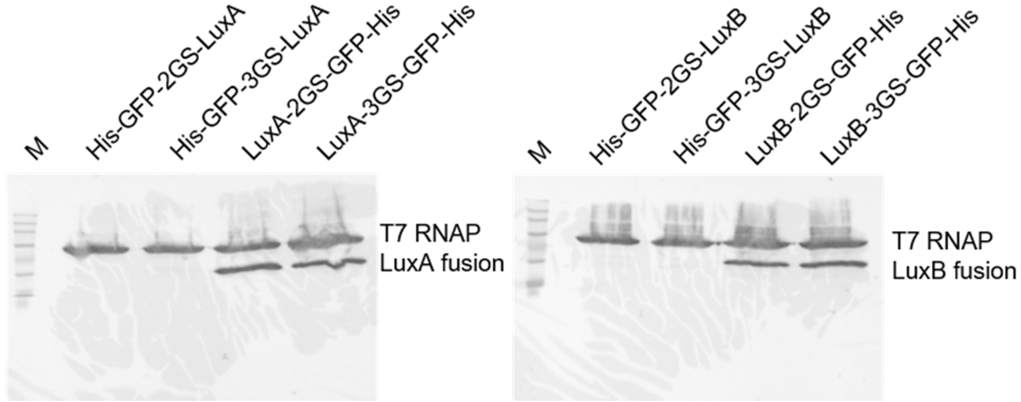

Figure S12 Western blot of LuxA- and LuxB-fusion proteins.

15 µl of TXTL was loaded on each lane and fractionated on a 7.5% gel for 60 minutes at 100V. The lane labeled with M stands for BLUEstain 2 Protein ladder, 5-245 kDa (Goldbio, P008-500). The protein names used for lane labels represent the fusion protein constructs from N-terminal to C-terminal order. His, His-Tag; GFP, enhanced green fluorescent protein; 2GS, two GS-linker sequence (GGGGS) repeats; 3GS, three GS-linker sequence repeats; T7 RNAP, T7 RNA polymerase. The bottom images are the original data.

Figure S13

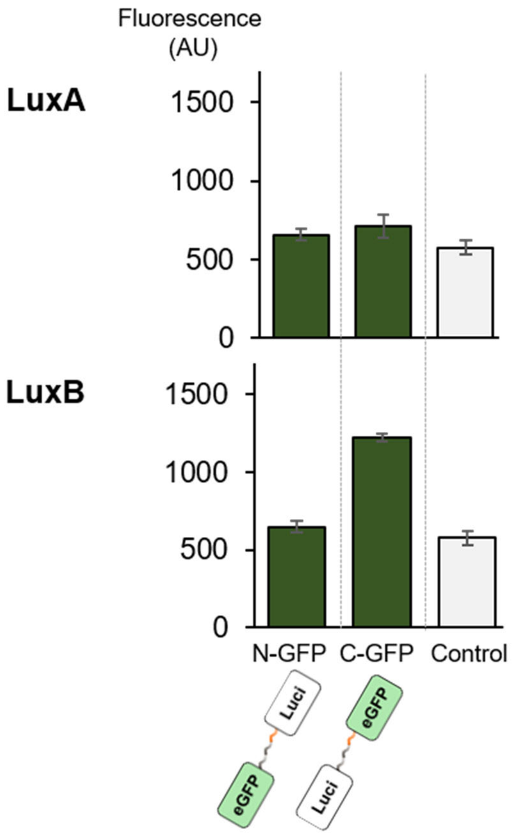

Figure S13 Fluorescence generated from eGFP-luciferase fusion proteins (LuxA and LuxB) with extended GS-linker.

All fusion proteins were expressed in TXTL at 30 ˚C for 8 hours, followed by fluorescence measurement. 19 µl of TXTL was used for the measurement. eGFP and luciferases are linked through 3x GS-linker (GGGGS.) Control stands for a reaction without protein expression.

Figure S14

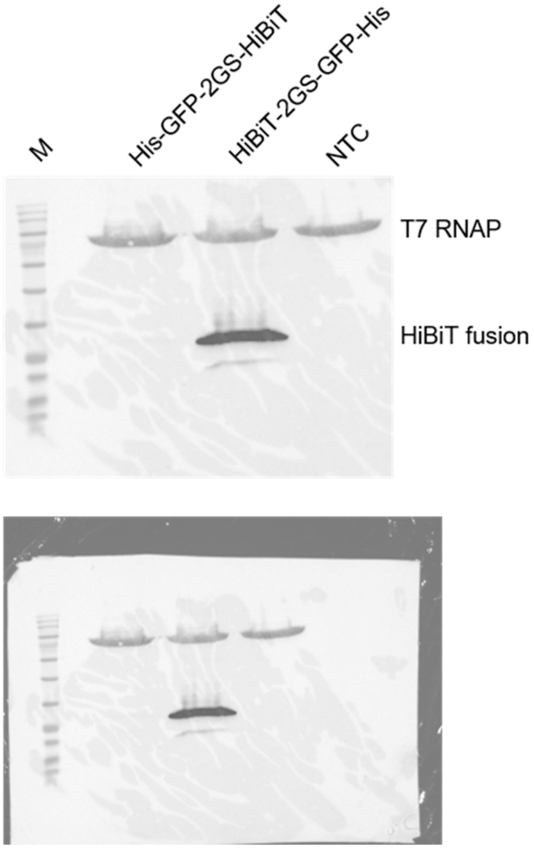

Figure S14 Western blot of HiBiT-fusion proteins.

15 µl of TXTL was loaded on each lane and fractionated on 7.5% gel for 60 minutes at 100V. The lane labeled with M stands for BLUEstain 2 Protein ladder, 5-245 kDa (Goldbio, P008-500). The protein names used for lane labels represent the protein structure from N-terminal to C-terminal order. His, His-Tag; GFP, enhanced green fluorescent protein; 2GS, two GS-linker sequence (GGGGS) repeats; T7 RNAP, T7 RNA polymerase. The bottom image is the original data.

Figure S15

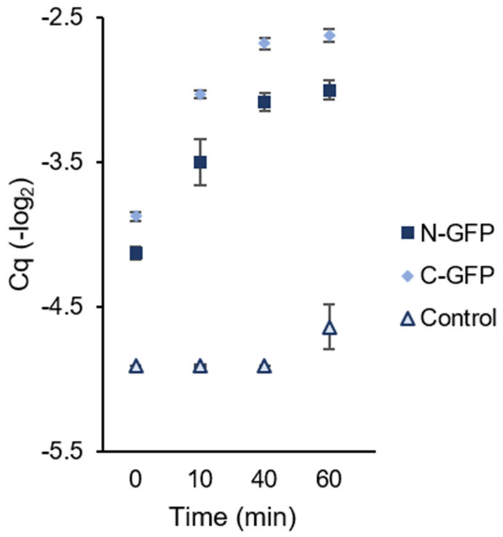

Figure S15 HiBiT-eGFP mRNA abundance in TXTL
RT-qPCR was performed to monitor the HiBiT fused eGFP mRNA level during the **Fig. 6D** kinetic reactions. The graphs show means with error bars that signify SEM (n = 3).

Figure S16

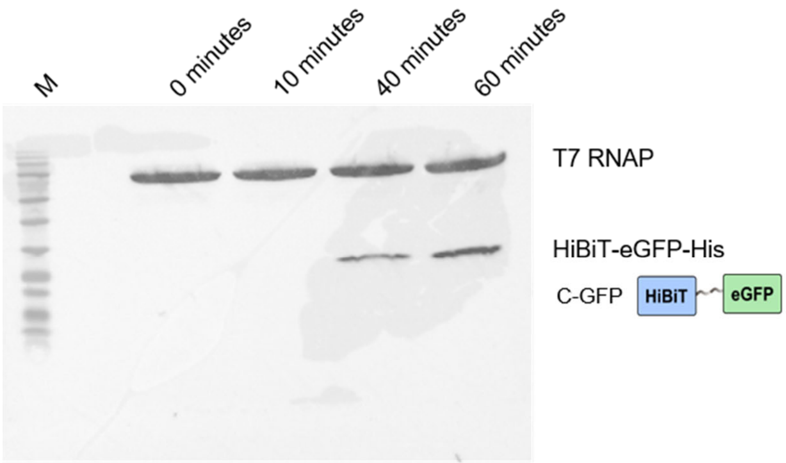

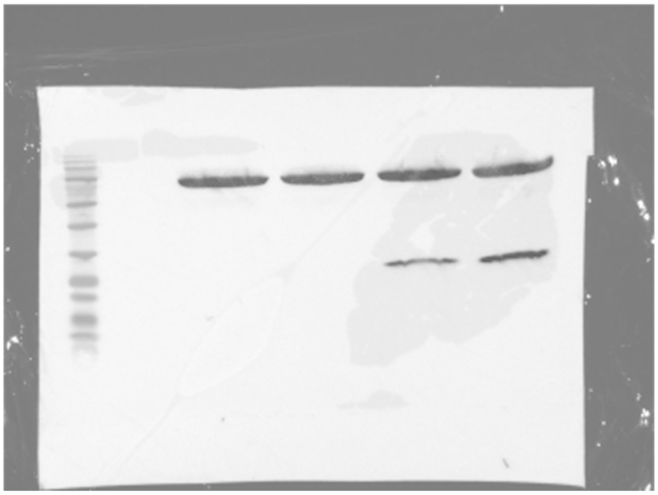

Figure S16 HiBiT-eGFP fusion protein abundance in TXTL.
Western blot of C-term eGFP fused HiBiT expressing TXTL during the **Fig. 6D** kinetic reactions**.** 15 µl of TXTL was loaded on each lane and fractionated on a 12% gel for 90 minutes at 100V. The lane labeled with M stands for BLUEstain 2 Protein ladder, 5-245 kDa (Goldbio, P008-500). The bottom image is the original data.

Figure S17

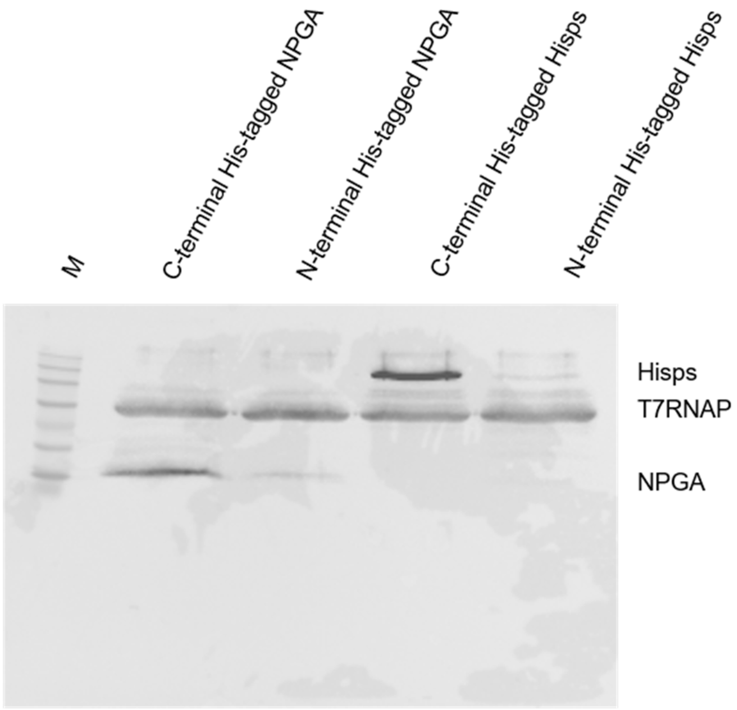

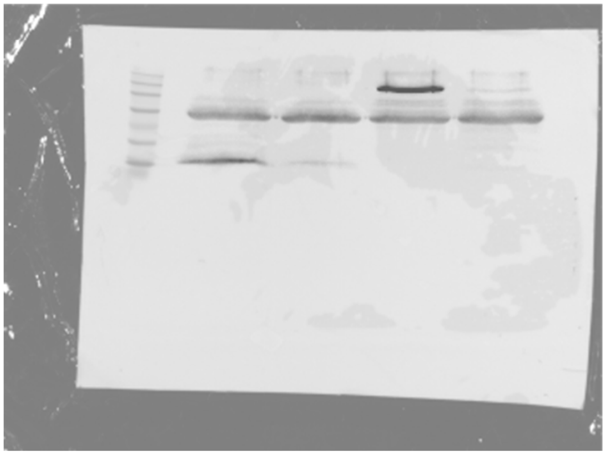

Figure S17 Fungal enzymes (NPGA and Hisps) expression in TXTL

10 µl of TXTL was loaded on each lane and fractionated on 7.5% gel for 70 minutes at 100V. The left lane labeled with M stands for BLUEstain 2 Protein ladder, 5-245 kDa (Goldbio, P008-500). The other lanes were labeled with the protein names that were expressed in the TXTL. NPGA, 4'-phosphopantetheinyl transferase; Hisps, hispidin synthase; T7 RNAP, T7 RNA polymerase. The bottom image is the original data.

Figure S18

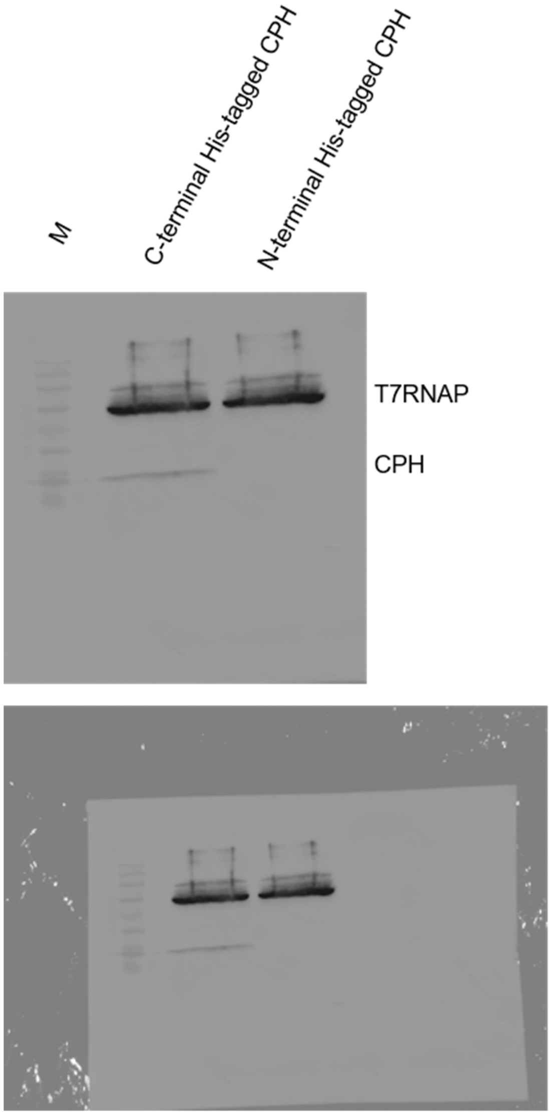

Figure S18 CPH expression in TXTL

10 µl of TXTL was loaded on each lane and fractionated on 7.5% gel for 70 minutes at 100V. The left lane labeled with M stands for BLUEstain 2 Protein ladder, 5-245 kDa (Goldbio, P008-500). The other lanes were labeled with the protein names that were expressed in the TXTL. CPH, caffeylpyruvate hydrolase; T7 RNAP, T7 RNA polymerase. The bottom image is the original data.

Figure S19

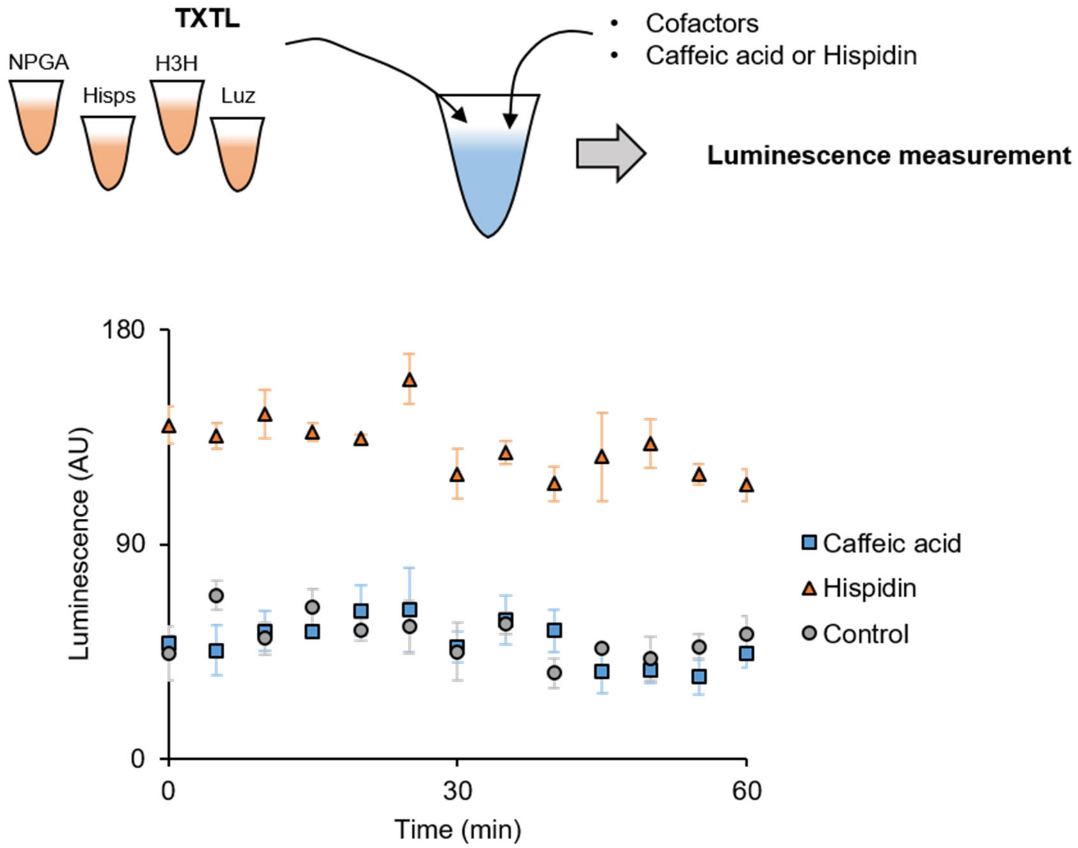

Figure S19 The result of caffeic acid conversion assay 1

NPGA, Hisps, H3H, and Luz were individually expressed in TXTL and mixed with cofactors and substrates (caffeic acid or hispidin). The luminescence was measured at 25˚C for 1 hour, every 5 minutes. While the reaction with hispidin generated light, the reaction with caffeic acid did not. This indicates that the caffeic acid was not converted into hispidin. Control contains TXTLs without enzyme expression.

Figure S20

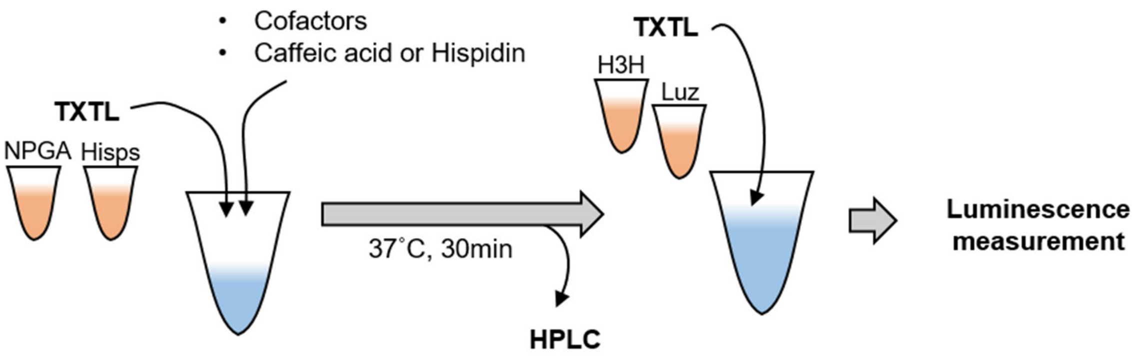

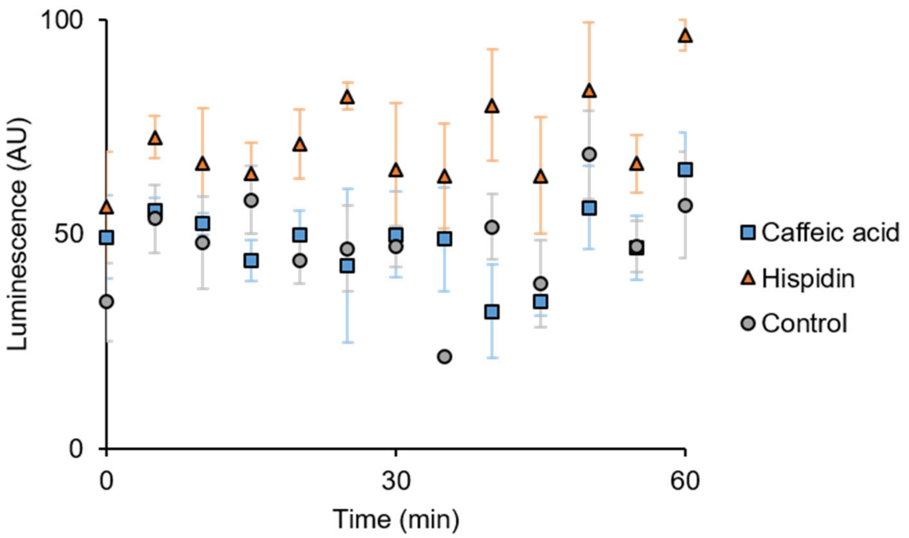

Figure S20 The result of caffeic acid conversion assay 2.
NPGA, Hisps, H3H, and Luz were individually expressed in TXTL. NPGA and Hisps TXTLs were first mixed with cofactors and substrates (caffeic acid or hispidin). The reaction was incubated at 37˚C for 30 minutes to facilitate phosphopantheteinylation. The aliquot of the reaction was taken for HPLC analysis after this incubation. Then, H3H and Luz TXTLs were added to the reaction and measured the luminescence at 25˚C for 1 hour, every 5 minutes. The reaction with hispidin slightly generated light. The reaction with caffeic acid was as same as Control reaction. This indicates that the caffeic acid was not converted into hispidin. Control stands for a reaction contains TXTLs without enzyme expression.

Figure S21

Blots for FLuc reaction

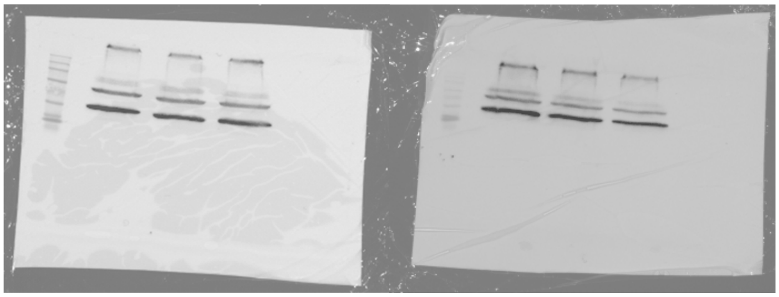

Blots for Rluc reaction

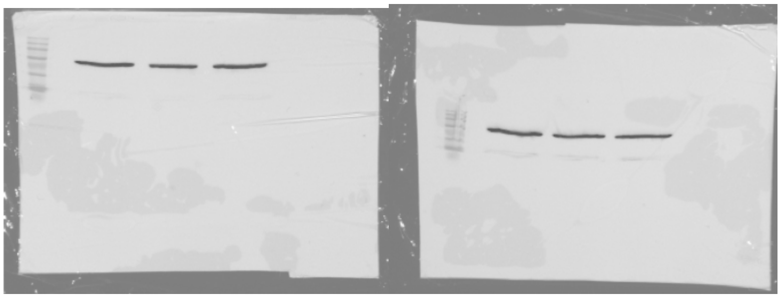

Blots for NanoLuc reaction

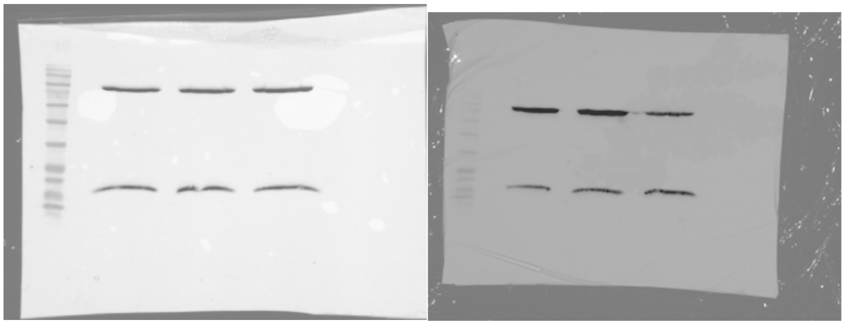

Blots for H3H-Luz

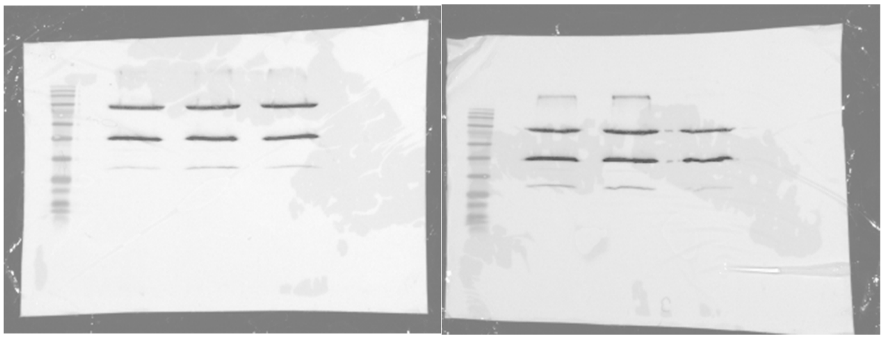

Figure S21 Original blots used in Fig. S1

Cloning methods

Primer sequences are listed in **Table S1**. Gene sequences are listed in **Table S2**. Plasmid names constructed in this paper are summarized in **Table S3**. Plasmids named with pTXTL contain a carbenicillin resistance gene and plasmids named with pLumi contain an ampicillin resistance gene for antibiotic selection. All the cloned plasmid sequences were verified by sequencing.

pTXTL-T7max-H3H, pTXTL-T7max-Luz, pTXTL-T7max-LuxA, pTXTL-T7max-LuxB, pTXTL-T7max-LuxC, pTXTL-T7max-LuxD, pTXTL-T7max-LuxE, pTXTL-T7max-Fre, pTXTL-T7max-FLuc, pTXTL-T7max-RLuc, and pTXTL-T7max-NanoLuc cloning.

The vector plasmid, pCI-T7Max-UTR1-CTerminus8xHis-T500, was digested with restriction enzymes at 37°C for 1 hour in 1x CutSmart Buffer (NEB, B7204). SpeI-HF (NEB, R3133L) and MluI-HF (NEB, R3198L) were used for H3H, Luz, Fre, FLuc, RLuc, and NanoLuc cloning. SpeI-HF and XhoI (NEB, R0146S) were used for LuxA, LuxB, LuxC, LuxD, and LuxE cloning. The digested plasmids were agarose gel purified using GenCatch Gel Extraction Kit (Epoch life science, 2260250). The purified vectors were treated with shrimp alkaline phosphatase (NEB, M0371L) at 37°C for 30 minutes, followed by the deactivation at 65°C for 15 minutes. Individual genes were amplified by Q5 High-Fidelity DNA Polymerase Master Mix (NEB, M0492L). H3H was amplified with P307-FBP_6, Primer1, and Primer2. Luz gene was amplified with gblock1, Primer3, and Primer4. Fre gene was amplified with gblock2, Primer15, and Primer16. FLuc and RLuc genes were amplified with pGreen_dualluc_3'UTR_sensor and primers (FLuc primers: Primer17 and Primer18, RLuc primers: Primer19 and Primer20). NanoLuc gene was amplified with pUAS-NanoLuc, Primer21, and Primer22. LuxA, LucB, LuxC, LuxD, and LuxB genes were amplified with pGEN-luxCDABE and primers (LuxA primers: Primer5 and Primer6, LuxB primers: Primer7 and Primer8, LuxC primers: Primer9 and Primer10, LuxD primers: Primer11 and Primer12, and LuxE primers: Primer13 and Primer14).

The PCR amplified genes were gel purified with GenCatch Gel Extraction Kit followed by restriction enzyme digestion, which the enzymes were the same as those that were used for vector digestion. The digested products were purified with GenCatch PCR Cleanup Kit (Epoch life science, 2360250). The vector and genes were ligated by T4 DNA Ligase (NEB, M0202L) in 1x T4 DNA Ligase reaction buffer (NEB, B0202S) at 16°C overnight. The following day, the ligase was deactivated at 65°C for 15 minutes and then transformed into *E. coli*.

pTXTL-T7max-HisGFPLuz, pTXTL-T7max-LuzGFPHis, and pLumi-T7max-H3H cloning.

PCR reactions with Q5 High-Fidelity DNA Polymerase Master Mix were prepared to produce PCR fragments. For pTXTL-T7max-HisGFPLuz, (1) N-term-His-eGFP sequence was amplified with Primer23 and Primer24, (2) Luz sequence was amplified with Primer25 and Primer26, and (3) the backbone sequence, pCI-T7Max-UTR1-NTerminus8xHis-T500, was amplified with Primer27 and Primer28. For pTXTL-T7max-LuzGFPHis, (1) C-term-His-eGFP sequence was amplified with Primer29 and Primer30, and (2) Luz sequence was amplified with Primer31 and Primer32. For pLumi-T7max-H3H, (1) the backbone sequence, pGEN-luxCDABE, was amplified with Primer33 and Primer34, and (2) H3H was amplified with Primer35 and Primer36. The PCR products were purified with GenCatch PCR Cleanup Kit. The assembly was performed by circular polymerase extension cloning (CPEC) reaction, which was previously described[32], then transformed into *E. coli*.

pTXTL-T7max-HisGFPHiBiT, pTXTL-T7max-HiBiTGFPHis, and pLumi-T7max-LgBiT cloning.
PCR reactions with 1x Q5 High-Fidelity DNA Polymerase Master Mix were prepared to produce PCR fragments. For pTXTL-T7max-HisGFPHiBiT, (1) pTXTL-T7max-HisGFPLuz sequence was amplified with Primer37 and Primer38, and (2) pTXTL-T7max-HisGFPLuz was amplified with Primer39 and Primer40. For pTXTL-T7max-HiBiTGFPHis, (1) pTXTL-T7max-LuzGFPHis was amplified with Primer41 and Primer37, and (2) pTXTL-T7max-LuzGFPHis was amplified with Primer42 and Primer40. For pLumi-T7max-LgBiT, (1) pBad-LgBiT-PhoCl1-SmBiT-MBP was amplified with Primer43 and Primer44, and (2) pGEN-luxCDABE was amplified with Priemr45 and Primer33, and (3) pGEN-luxCDABE was amplified with Primer46 and Primer34. The PCR products were purified with GenCatch PCR Cleanup Kit. The assembly was performed with Gibson Assembly 1x Master Mix (NEB, E2611L) and incubated at 50°C for 30 minutes. The assembled products were transformed into *E. coli*.

Efforts to engineer the fungi luciferase substrate regeneration pathway

NPGA, Hisps, and CPH are required in addition to Luz-H3H system for reconstructing the fungi substrate regeneration pathway (**Fig. S6**). First, we cloned NPGA, Hisps, and CPH genes into a vector plasmid, pCI-T7Max-UTR1-CTerminus8xHis-T500 or pCI-T7Max-UTR1-NTerminus8xHis-T500[24]. Those genes' expressions were confirmed by Western blot (**Fig. S17, S18**). We used C-terminal His-tag constructs for NPGA, Hisps, and CPH, in later experiments, because of more robust expression in TXTL (**Fig. S17, S18**).

First, we expressed NPGA, Hisps, H3H, and Luz individually in 4 different TXTLs at 30˚C for 8 hours. Then, in 90 µl reaction, we mixed the following components: 20 µl of each TXTLs, 2 mM ATP, 2 mM malonyl CoA, 1 mM CoA, and 1 mM NADPH. For substrate, we added 2 mM Caffeic acid or 200 µM Hispidin. We incubated the reaction at 25˚C and measured the luminescence for 1 hour, every 5 minutes. However, only Hispidin-containing reaction produced luminescence (**Fig. S19**).

In a previous report, *in vitro* phosphopantheteinylation assay with NPGA was performed at 37˚C, instead of 30˚C (our TXTL reaction)[33]. Thus, we split the reaction into two steps: (1) Conversion of caffeic acid to hispidin and (2) light generative reaction from hispidin. Briefly, we expressed NPGA, Hisps, H3H, and Luz individually at 30˚C for 8 hours. Then, we mixed NPGA and Hisps TXTLs with 2 mM ATP, 2 mM malonyl CoA, 1 mM CoA, 1 mM NADPH, and 10 mM $MgCl_2$. $MgCl_2$ was added to make the reaction condition similar to the previous *in vitro* assay[33]. The reaction was incubated at 37˚C for 30 minutes. The aliquot of samples was analyzed by high-performance liquid chromatography (HPLC); however, we did not detect the conversion from caffeic acid to hispidin. We then added H3H and Luz TXTLs to the reaction mixture and measured the luminescence at 25˚C for 1 hour, every 5 minutes. We observed slight luminescence from a reaction containing hispidin as the substrate; however, the reaction containing caffeic acid did not generate any light (**Fig. S20**).

Lastly, we purified both NPGA and Hisps through His-tagged protein purification and performed an *in vitro* assay to see the conversion from caffeic acid to hispidin. The 40 µl reaction contained 100 mM Tris-HCl buffer (pH6.8), 1 µM NPGA, 1 µM Hisps, 2 mM ATP, 2 mM malonyl CoA, 500 µM CoA, and 40 mM $MgCl_2$. The substrate was either 2 mM caffeic acid or 200 µM hispidin. The reaction was incubated at 37˚C for 30 minutes. After the incubation, we stopped the reaction by adding 200 µl of ethanol, and the liquid was evaporated completely by a speed vacuum concentrator. The pellets were dissolved in methanol to inject HPLC. However, we could not detect the conversion of caffeic acid to hispidin by HPLC.

Table S1 Primer sequences

Name	Sequence
Primer1	AGCATACTAGTATGGCATCCTTTGAAAATTCACTCA
Primer2	CTGTAACGCGTAGCTGAGTTGGATGATCGC
Primer3	AGCATACTAGTATGCGTATCAACATCTCCC
Primer4	CTGTAACGCGTTTTGGCATTCTCGACGAT
Primer5	AGCATACTAGTATGAAATTTGGAAACTTTTTGCTTACATACCAA
Primer6	TACAGCTCGAGTTAATATAATAGCGAACGTTGTTTTCTTTAAGAAATGG
Primer7	AGCATACTAGTATGAAATTTGGATTGTTCTTCCTTAACTTCATC
Primer8	TACAGCTCGAGTTAGGTATATTCCATGTGGTACTTCTTAATATTATCATCAA
Primer9	AGCATACTAGTATGACTAAAAAAATTTCATTCATTATTAACGGCCAG
Primer10	TACAGCTCGAGTTATGGGACAAATACAAGGAACTTATCTTCTT
Primer11	AGCATACTAGTATGGAAAATGAATCAAAATATAAAACCATCGACC
Primer12	TACAGCTCGAGTTAAGACAGAGAAATTGCTTGATTTTCAATCTC
Primer13	AGCATACTAGTATGACTTCATATGTTGATAAACAAGAAATTACAGC
Primer14	TACAGCTCGAGTTAACTATCAAACGCTTCGGTTAAGC
Primer15	AGCATACTAGTATGACGACCCTGTCCTGTAAA
Primer16	CCTGTAACGCGTGATAAACGCGAACGCATCAC
Primer17	AGCATACTAGTATGGAAGACGCCAAAAACATAAAGAAA
Primer18	CCTGTAACGCGTCACGGCGATCTTTCCGC
Primer19	AGCATACTAGTATGACTTCGAAAGTTTATGATCCAGAAC
Primer20	CCTGTAACGCGTTTGTTCATTTTTGAGAACTCGCTCAA
Primer21	AGCATACTAGTATGGTCTTCACACTCGAAGATTTC
Primer22	CCTGTAACGCGTCGCCAGAATGCGTTCGC
Primer23	ACTAGTATGGAGCTTTTCACTGG
Primer24	TGATCCCCCTCCGCCCGATCCACCACCTCCGATCCCGGCGGC
Primer25	GCGGAGGGGGATCAATGCGTATCAACATCTCCCTG
Primer26	GCTCGAGTTAACGCGTTTATTTGGCATTCTCGACGATTTTACC
Primer27	ACGCGTTAACTCGAGCAAA
Primer28	CCAGTGAAAAGCTCCATACTAGTGCCACCTCCGTGGT
Primer29	GCGGAGGGGGATCAATGGAGCTTTTCACTGGCG
Primer30	TTATCCGTGGTGATGGTGATG
Primer31	CATCACCATCACCACGGATAACTCGAGCAAAGCCCG
Primer32	TGATCCCCCTCCGCCCGATCCACCACCTCCTTTGGCATTCTCGACGATTTTACC
Primer33	TGACCTGCAGGCATGC
Primer34	ACGTATCCTCCAAGCCTGA
Primer35	TCAGGCTTGGAGGATACGTATGGCATCCTTTGAAAATTCACTCA
Primer36	CATGCCTGCAGGTCAAGCTGAGTTGGATGATCGC

Primer37	TTCTGACAACGATCGGAGGA
Primer38	AGCCGCCAGCCGCTCACCATTGATCCCCCTCCGC
Primer39	ATGGTGAGCGGCTGGCGGCTGTTCAAGAAGATTAGCTAACTCGAGCAAAGCCCG
Primer40	TCCTCCGATCGTTGTCAGAA
Primer41	AACAGCCGCCAGCCGCTCACCATACTAGTCATACCGGTATATCTCCTTCTTA
Primer42	GTGAGCGGCTGGCGGCTGTTCAAGAAGATTAGCGGAGGTGGTGGATCGG
Primer43	TTCAGGCTTGGAGGATACGTATGGTCTTCACACTCGAAGATTT
Primer44	GCTTGCATGCCTGCAGGTCAGCTGTTGATGGTTACTCGGAA
Primer45	CCGCTTTTTTGCACAACATGG
Primer46	CCATGTTGTGCAAAAAAGCGG

Table S2 Gene sequence information

gblock1, Luz gene	ATGCGTATAAATATCTCTCTGTCTAGCTTATTCGAGAGATTAAGCAAACTCAGCTCTCGTAGCATA<br>GCTATTACATGTGGTGTCGTCCTCGCCTCTGCTATTGCTTTTCCCATCATACGACGAGATTATCAGA<br>CGTTCTTAGAGGTTGGTCCTAGCTATGCCCCTCAAAATTTTCGAGGCTACATTATCGTTTGCGTACT<br>CAGCTTGTTCCGACAAGAGCAAAAAGGGTTGGCAATCTACGACAGACTCCCAGAGAAGAGAAGA<br>TGGCTGGCCGATCTCCCATTTCGTGAAGGTACTAGACCTAGTATAACATCACACATAATACAGAG<br>ACAGCGAACTCAACTTGTAGACCAAGAGTTTGCCACACGAGAACTGATAGACAAAGTTATTCCCA<br>GGGTGCAAGCACGACATACAGATAAAACATTTTTGAGCACGAGCAAGTTTGAATTTCATGCTAAA<br>GCCATTTTCCTGTTGCCAAGTATTCCTATAAACGACCCCTTAAACATACCTAGTCATGATACGGTTC<br>GTAGAACGAAACGAGAGATTGCACACATGCATGACTACCACGACTGTACTCTTCACTTAGCTCTT<br>GCAGCTCAAGATGGAAAAGAAGTCCTGAAAAAAGGCTGGGGCCAACGTCATCCGCTTGCCGGGC<br>CGGGTGTACCTGGGCCGCCGACAGAATGGACTTTCTTATACGCACCCCGTAATGAGGAAGAAGC<br>TAGGGTAGTAGAAATGATTGTCGAGGCAAGTATCGGATACATGACTAATGATCCGGCAGGTAAA<br>ATCGTCGAGAATGCCAAATGA
gblock2, Fre gene	ATGACGACCCTGTCCTGTAAAGTTACGAGCGTAGAAGCGATCACGGATACCGTCTACCGCGTGAG<br>AATAGTCCCTGATGCTGCCTTCAGTTTTAGGGCGGGGCAGTACCTAATGGTTGTGATGGATGAAC<br>GCGACAAACGACCGTTTAGCATGGCGAGTACCCCGGACGAGAAGGGCTTTATTGAACTGCATATT<br>GGCGCATCAGAAATCAATTTATATGCAAAAGCTGTGATGGACCGCATTCTGAAAGATCACCAGAT<br>TGTCGTTGATATCCCGCATGGCGAAGCATGGCTGCGGGATGATGAAGAGCGTCCAATGATTCTTA<br>TCGCTGGTGGCACTGGTTTCAGCTACGCCCGGTCGATATTGCTCACAGCGCTGGCCCGTAACCCG<br>AACCGTGATATTACCATCTATTGGGGGGGTCGTGAAGAACAACACTTGTATGATTTGTGCGAGCT<br>GGAGGCCCTGTCGCTGAAGCATCCCGGCCTGCAGGTAGTGCCGGTAGTGGAACAACCAGAGGCT<br>GGGTGGCGTGGACGCACTGGCACAGTTCTTACCGCAGTGCTCCAGGACCATGGAACGTTAGCCG<br>AACACGACATTTATATTGCCGGTCGCTTTGAGATGGCAAAAATCGCGCGCGATTTATTTTGCTCTG<br>AACGTAATGCGCGCGAAGACCGTCTGTTCGGTGATGCGTTCGCGTTTATC

H3H	ATGGCATCCTTTGAAAATTCACTCAGTGTCTTAATAGTTGGGGCTGGCTTAGGTGGTTTGGCCGCT<br>GCTATAGCACTGCGAAGGCAAGGACATGTAGTCAAGATATACGATAGTTCTTCATTCAAGGCCGA<br>ATTGGGAGCAGGATTAGCTGTACCACCCAATACTCTCCGTTCCCTGCAACAACTGGGCTGTAATAC<br>AGAAAACCTGAACGGAGTAGACAACCTCTGCTTTACGGCCATGGGATACGACGGGAGTGTAGGT<br>ATGATGAATAATATGACCGACTACCGTGAGGCATACGGCACTTCCTGGATAATGGTACATCGAGT<br>GGATCTCCACAACGAGCTGATGCGAGTCGCCTTGGATCCGGGCGGGTTAGGGCCTCCTGCCACTC<br>TTCACCTTAACCATCGTGTTACGTTCTGCGATGTGGATGCCTGTACAGTCACGTTTACGAACGGGA<br>CGACACAGTCTGCTGACTTGATTGTCGGAGCTGATGGTATTAGGTCTACGATTCGAAGATTCGTTC<br>TGGAGGAGGACGTGACTGTTCCAGCTAGCGGAATTGTCGGCTTTCGATGGTTGGTCCAGGCCGA<br>TGCTCTCGACCCATATCCCGAACTGGATTGGATTGTCAAAAAACCACCCTTGGGAGCCAGGTTGA<br>TTTCTACTCCACAGAACCCGCAGAGTGGCGTAGGACTGGCCGATAGACGAACGATCATTATTTAC<br>GCATGCAGAGGTGGGACCATGGTTAATGTATTAGCCGTACACGACGACGAAAGAGATCAAAACA<br>CTGCTGATTGGTCCGTTCCCGCATCAAAGGATGATTTATTCAGAGTGTTTCACGATTACCATCCAA<br>GATTCCGACGACTCTTAGAGTTAGCACAGGACATTAATTTATGGCAGATGAGGGTGGTCCCGGTC<br>TTGAAAAAATGGGTGAACAAGAGGGTATGCTTGCTGGGTGATGCCGCCCATGCTTCACTGCCCAC<br>ACTTGGACAAGGCTTCGGGATGGGGTTGGAGGATGCAGTCGCTTTGGGCACGTTGTTGCCTAAG<br>GGAACGACCGCATCACAGATCGAGACAAGGCTGGCCGTTTACGAACAGCTGCGAAAGGATCGAG<br>CTGAGTTTGTCGCCGCAGAGAGCTATGAGGAGCAATATGTCCCAGAGATGAGAGGGCTGTACCT<br>TAGGTCCAAAGAATTGAGAGACAGAGTGATGGGTTATGATATTAAGGTAGAGTCAGAGAAGGTT<br>TTGGAAACACTGTTGCGATCATCCAACTCAGCT
Luz	ATGCGTATCAACATCTCCCTGTCGTCGTTATTCGAACGGTTAAGTAAACTGAGCTCTCGTTCAATT<br>GCGATTACCTGTGGAGTTGTCCTCGCCTCTGCTATTGCTTTTCCCATCATACGTCGGGATTATCAG<br>ACGTTCCTGGAGGTTGGTCCTAGCTATGCCCCGCAAAACTTTCGCGGCTACATTATCGTTTGCGTA<br>CTTAGCTTGTTCCGCCAAGAGCAGAAAGGCCTGGCGATCTACGACCGGCTCCCAGAGAAGCGCC<br>GCTGGCTGGCGGATCTGCCATTTCGTGAAGGTACCAGACCGAGTATTACCTCACACATTATCCAG<br>CGCCAGCGCACTCAACTTGTGGATCAGGAATTTGCCACACGTGAACTGATTGACAAGGTTATTCC<br>GAGGGTGCAAGCACGACATACCGATAAAACATTTTTGAGCACGAGCAAGTTTGAATTTCATGCGA<br>AAGCCATTTTCCTGTTGCCATCGATTCCAATCAACGACCCGCTGAACATACCTAGTCATGATACGG<br>TGCGTCGAACGAAACGCGAAATTGCCCACATGCATGACTATCACGATTGCACCCTTCACCTAGCTC<br>TGGCGGCACAGGATGGAAAAGAAGTCCTGAAAAAAGGCTGGGGCCAGCGTCATCCGCTGGCGG<br>GGCCGGGTGTGCCTGGGCCGCCGACTGAATGGACTTTCTTATACGCACCCCGTAATGAGGAAGA<br>AGCGCGCGTGGTAGAAATGATTGTGGAAGCATCCATCGGCTATATGACCAATGATCCGGCAGGT<br>AAAATCGTCGAGAATGCCAAA

LuxA	ATGAAATTTGGAAACTTTTTGCTTACATACCAACCTCCCCAATTTTCTCAAACAGAGGTAATGAAA<br>CGTTTGGTTAAATTAGGTCGCATCTCTGAGGAGTGTGGTTTTGATACCGTATGGTTACTGGAGCA<br>TCATTTCACGGAGTTTGGTTTGCTTGGTAACCCTTATGTCGCTGCTGCATATTTACTTGGCGCGACT<br>AAAAAATTGAATGTAGGAACTGCCGCTATTGTTCTTCCCACAGCCCATCCAGTACGCCAACTTGAA<br>GATGTGAATTTATTGGATCAAATGTCAAAAGGACGATTTCGGTTTGGTATTTGCCGAGGGCTTTA<br>CAACAAGGACTTTCGCGTATTCGGCACAGATATGAATAACAGTCGCGCCTTAGCGGAATGCTGGT<br>ACGGGCTGATAAAGAATGGCATGACAGAGGGATATATGGAAGCTGATAATGAACATATCAAGTT<br>CCATAAGGTAAAAGTAAACCCCGCGGCGTATAGCAGAGGTGGCGCACCGGTTTATGTGGTGGCT<br>GAATCAGCTTCGACGACTGAGTGGGCTGCTCAATTTGGCCTACCGATGATATTAAGTTGGATTAT<br>AAATACTAACGAAAAGAAAGCACAACTTGAGCTTTATAATGAAGTGGCTCAAGAATATGGGCAC<br>GATATTCATAATATCGACCATTGCTTATCATATATAACATCTGTAGATCATGACTCAATTAAAGCGA<br>AAGAGATTTGCCGGAAATTTCTGGGGCATTGGTATGATTCTTATGTGAATGCTACGACTATTTTTG<br>ATGATTCAGACCAAACAAGAGGTTATGATTTCAATAAAGGGCAGTGGCGTGACTTTGTATTAAAA<br>GGACATAAAGATACTAATCGCCGTATTGATTACAGTTACGAAATCAATCCCGTGGGAACGCCGCA<br>GGAATGTATTGACATAATTCAAAAAGACATTGATGCTACAGGAATATCAAATATTTGTTGTGGATT<br>TGAAGCTAATGGAACAGTAGACGAAATTATTGCTTCCATGAAGCTCTTCCAGTCTGATGTCATGCC<br>ATTTCTTAAAGAAAAACAACGTTCGCTATTATAT
LuxB	ATGAAATTTGGATTGTTCTTCCTTAACTTCATCAATTCAACAACTGTTCAAGAACAAAGTATAGTTC<br>GCATGCAGGAAATAACGGAGTATGTTGATAAGTTGAATTTTGAACAGATTTTAGTGTATGAAAAT<br>CATTTTTCAGATAATGGTGTTGTCGGCGCTCCTCTGACTGTTTCTGGTTTTCTGCTCGGTTTAACAG<br>AGAAAATTAAAATTGGTTCATTAAATCACATCATTACAACTCATCATCCTGTCCGCATAGCGGAGG<br>AAGCTTGCTTATTGGATCAGTTAAGTGAAGGGAGATTTATTTTAGGGTTTAGTGATTGCGAAAAA<br>AAAGATGAAATGCATTTTTTTAATCGCCCGGTTGAATATCAACAGCAACTATTTGAAGAGTGTTAT<br>GAAATCATTAACGATGCTTTAACAACAGGCTATTGTAATCCAGATAACGATTTTTATAGCTTCCCT<br>AAAATATCTGTAAATCCCCATGCTTATACGCCAGGCGGACCTCGGAAATATGTAACAGCAACCAG<br>TCATCATATTGTTGAGTGGGCGGCCAAAAAAGGTATTCCTCTCATCTTTAAGTGGGATGATTCTAA<br>TGATGTTAGATATGAATATGCTGAAAGATATAAAGCCGTTGCGGATAAATATGACGTTGACCTAT<br>CAGAGATAGACCATCAGTTAATGATATTAGTTAACTATAACGAAGATAGTAATAAAGCTAAACAA<br>GAGACGCGTGCATTTATTAGTGATTATGTTCTTGAAATGCACCCTAATGAAAATTTCGAAAATAAA<br>CTTGAAGAAATAATTGCAGAAAACGCTGTCGGAAATTATACGGAGTGTATAACTGCGGCTAAGTT<br>GGCAATTGAAAAGTGTGGTGCGAAAAGTGTATTGCTGTCCTTTGAACCAATGAATGATTTGATGA<br>GCCAAAAAAATGTAATCAATATTGTTGATGATAATATTAAGAAGTACCACATGGAATATACC

LuxC	ATGACTAAAAAAATTTCATTCATTATTAACGGCCAGGTTGAAATCTTTCCCGAAGGTGATGATTTA<br>GTGCAATCCATTAATTTTGGTGATAATAGTGTTTACCTGCCAATATTGAATGACTCTCATGTAAAA<br>AACATTATTGATTGTAATGGAAATAACGAATTACGGTTGCATAACATTGTCAATTTTCTCTATACG<br>GTAGGGCAAAGATGGAAAAATGAAGAATACTCAAGACGCAGGACATACATTCGTGACTTAAAAA<br>AATATATGGGATATTCAGAAGAAATGGCTAAGCTAGAGGCCAATTGGATATCTATGATTTTATGT<br>TCTAAAGGCGGCCTTTATGATGTTGTAGAAAATGAACTTGGTTCTCGCCATATCATGGATGAATG<br>GCTACCTCAGGATGAAAGTTATGTTCGGGCTTTTCCGAAAGGTAAATCTGTACATCTGTTGGCAG<br>GTAATGTTCCATTATCTGGGATCATGTCTATATTACGCGCAATTTTAACTAAGAATCAGTGTATTAT<br>AAAAACATCGTCAACCGATCCTTTTACCGCTAATGCATTAGCGTTAAGTTTTATTGATGTAGACCCT<br>AATCATCCGATAACGCGCTCTTTATCTGTTATATATTGGCCCCACCAAGGTGATACATCACTCGCA<br>AAAGAAATTATGCAACATGCGGATGTTATTGTCGCTTGGGGAGGGCCAGATGCGATTAATTGGG<br>CGGTAGAGCATGCGCCATCTTATGCTGATGTGATTAAATTTGGTTCTAAAAAGAGTCTTTGCATTA<br>TCGATAATCCTGTTGATTTGACGTCCGCAGCGACAGGTGCGGCTCATGATGTTTGTTTTTACGATC<br>AGCGAGCTTGTTTTTCTGCCCAAAACATATATTACATGGGAAATCATTATGAGGAATTTAAGTTAG<br>CGTTGATAGAAAAACTTAATCTATATGCGCATATATTACCGAATGCCAAAAAAGATTTTGATGAAA<br>AGGCGGCCTATTCTTTAGTTCAAAAAGAAAGCTTGTTTGCTGGATTAAAAGTAGAGGTGGATATT<br>CATCAACGTTGGATGATTATTGAGTCAAATGCAGGTGTGGAATTTAATCAACCACTTGGCAGATG<br>TGTGTACCTTCATCACGTCGATAATATTGAGCAAATATTGCCTTATGTTCAAAAAAATAAGACGCA<br>AACCATATCTATTTTTCCTTGGGAGTCATCATTTAAATATCGAGATGCGTTAGCATTAAAAGGTGC<br>GGAAAGGATTGTAGAAGCAGGAATGAATAACATATTTCGAGTTGGTGGATCTCATGACGGAATG<br>AGACCGTTGCAACGATTAGTGACATATATTTCTCATGAAAGGCCATCTAACTATACGGCTAAGGA<br>TGTTGCGGTTGAAATAGAACAGACTCGATTCCTGGAAGAAGATAAGTTCCTTGTATTTGTCCCA
LuxD	ATGGAAAATGAATCAAAATATAAAACCATCGACCACGTTATTTGTGTTGAAGGAAATAAAAAAAT<br>TCATGTTTGGGAAACGCTGCCAGAAGAAAACAGCCCAAAGAGAAAGAATGCCATTATTATTGCGT<br>CTGGTTTTGCCCGCAGGATGGATCATTTTGCTGGTCTGGCGGAATATTTATCGCGGAATGGATTTC<br>ATGTGATCCGCTATGATTCGCTTCACCACGTTGGATTGAGTTCAGGGACAATTGATGAATTTACAA<br>TGTCTATAGGAAAGCAGAGCTTGTTAGCAGTGGTTGATTGGTTAACTACACGAAAAATAAATAAC<br>TTCGGTATGTTGGCTTCAAGCTTATCTGCGCGGATAGCTTATGCAAGCCTATCTGAAATCAATGCT<br>TCGTTTTTAATCACCGCAGTCGGTGTTGTTAACTTAAGATATTCTCTTGAAAGAGCTTTAGGGTTTG<br>ATTATCTCAGTCTACCCATTAATGAATTGCCGAATAATCTAGATTTTGAAGGCCATAAATTGGGTG<br>CTGAAGTCTTTGCGAGAGATTGTCTTGATTTTGGTTGGGAAGATTTAGCTTCTACAATTAATAACA<br>TGATGTATCTTGATATACCGTTTATTGCTTTTACTGCAAATAACGATAATTGGGTCAAGCAAGATG<br>AAGTTATCACATTGTTATCAAATATTCGTAGTAATCGATGCAAGATATATTCTTTGTTAGGAAGTTC<br>GCATGACTTGAGTGAAAATTTAGTGGTCCTGCGCAATTTTTATCAATCGGTTACGAAAGCCGCTAT<br>CGCGATGGATAATGATCATCTGGATATTGATGTTGATATTACTGAACCGTCATTTGAACATTTAAC<br>TATTGCGACAGTCAATGAACGCCGAATGAGAATTGAGATTGAAAATCAAGCAATTTCTCTGTCT

LuxE	ATGACTTCATATGTTGATAAACAAGAAATTACAGCAAGCTCAGAAATTGATGATTTGATTTTTTCG<br>AGCGATCCATTAGTGTGGTCTTACGACGAGCAGGAAAAAATCAGAAAGAAACTTGTGCTTGATGC<br>ATTTCGTAATCATTATAAACATTGTCGAGAATATCGTCACTACTGTCAGGCACACAAAGTAGATGA<br>CAATATTACGGAAATTGATGACATACCTGTATTCCCAACATCGGTTTTTAAGTTTACTCGCTTATTA<br>ACTTCTCAGGAAAACGAGATTGAAAGTTGGTTTACCAGTAGCGGCACGAATGGTTTAAAAAGTCA<br>GGTGGCGCGTGACAGATTAAGTATTGAGAGACTCTTAGGCTCTGTGAGTTATGGCATGAAATATG<br>TTGGTAGTTGGTTTGATCATCAAATAGAATTAGTCAATTTGGGACCAGATAGATTTAATGCTCATA<br>ATATTTGGTTTAAATATGTTATGAGTTTGGTGGAATTGTTATATCCTACGACATTTACCGTAACAG<br>AAGAACGAATAGATTTTGTTAAAACATTGAATAGTCTTGAACGAATAAAAAATCAAGGGAAAGAT<br>CTTTGTCTTATTGGTTCGCCATACTTTATTTATTTACTCTGCCATTATATGAAAGATAAAAAAATCTC<br>ATTTTCTGGAGATAAAAGCCTTTATATCATAACCGGAGGCGGCTGGAAAAGTTACGAAAAAGAAT<br>CTCTGAAACGTGATGATTTCAATCATCTTTTATTTGATACTTTCAATCTCAGTGATATTAGTCAGAT<br>CCGAGATATATTTAATCAAGTTGAACTCAACACTTGTTTCTTTGAGGATGAAATGCAGCGTAAACA<br>TGTTCCGCCGTGGGTATATGCGCGAGCGCTTGATCCTGAAACGTTGAAACCTGTACCTGATGGAA<br>CGCCGGGGTTGATGAGTTATATGGATGCGTCAGCAACCAGTTATCCAGCATTTATTGTTACCGAT<br>GATGTCGGGATAATTAGCAGAGAATATGGTAAGTATCCCGGCGTGCTCGTTGAAATTTTACGTCG<br>CGTCAATACGAGGACGCAGAAAGGGTGTGCTTTAAGCTTAACCGAAGCGTTTGATAGT

FLuc	ATGGAAGACGCCAAAAACATAAAGAAAGGCCCGGCGCCATTCTATCCGCTGGAAGATGGAACCG<br>CTGGAGAGCAACTGCATAAGGCTATGAAGAGATACGCCCTGGTTCCTGGAACAATTGCTTTTACA<br>GATGCACATATCGAGGTGGACATCACTTACGCTGAGTACTTCGAAATGTCCGTTCGGTTGGCAGA<br>AGCTATGAAACGATATGGGCTGAATACAAATCACAGAATCGTCGTATGCAGTGAAAACTCTCTTC<br>AATTCTTTATGCCGGTGTTGGGCGCGTTATTTATCGGAGTTGCAGTTGCGCCCGCGAACGACATTT<br>ATAATGAACGTGAATTGCTCAACAGTATGGGCATTTCGCAGCCTACCGTGGTGTTCGTTTCCAAAA<br>AGGGGTTGCAAAAAATTTTGAACGTGCAAAAAAAGCTCCCAATCATCCAAAAAATTATTATCATG<br>GATTCTAAAACGGATTACCAGGGATTTCAGTCGATGTACACGTTCGTCACATCTCATCTACCTCCC<br>GGTTTTAATGAATACGATTTTGTGCCAGAGTCCTTCGATAGGGACAAGACAATTGCACTGATCAT<br>GAACTCCTCTGGATCTACTGGTCTGCCTAAAGGTGTCGCTCTGCCTCATAGAACTGCCTGCGTGAG<br>ATTCTCGCATGCCAGAGATCCTATTTTTGGCAATCAAATCATTCCGGATACTGCGATTTTAAGTGTT<br>GTTCCATTCCATCACGGTTTTGGAATGTTTACTACACTCGGATATTTGATATGTGGATTTCGAGTC<br>GTCTTAATGTATAGATTTGAAGAAGAGCTGTTTCTGAGGAGCCTTCAGGATTACAAGATTCAAAG<br>TGCGCTGCTGGTGCCAACCCTATTCTCCTTCTTCGCCAAAAGCACTCTGATTGACAAATACGATTTA<br>TCTAATTTACACGAAATTGCTTCTGGTGGCGCTCCCCTCTCTAAGGAAGTCGGGGAAGCGGTTGC<br>CAAGAGGTTCCATCTGCCAGGTATCAGGCAAGGATATGGGCTCACTGAGACTACATCAGCTATTC<br>TGATTACACCCGAGGGGGATGATAAACCGGGCGCGGTCGGTAAAGTTGTTCCATTTTTTGAAGCG<br>AAGGTTGTGGATCTGGATACCGGGAAAACGCTGGGCGTTAATCAAAGAGGCGAACTGTGTGTGA<br>GAGGTCCTATGATTATGTCCGGTTATGTAAACAATCCGGAAGCGACCAACGCCTTGATTGACAAG<br>GATGGATGGCTACATTCTGGAGACATAGCTTACTGGGACGAAGACGAACACTTCTTCATCGTTGA<br>CCGCCTGAAGTCTCTGATTAAGTACAAAGGCTATCAGGTGGCTCCCGCTGAATTGGAATCCATCTT<br>GCTCCAACACCCCAACATCTTCGACGCAGGTGTCGCAGGTCTTCCCGACGATGACGCCGGTGAAC<br>TTCCCGCCGCCGTTGTTGTTTTGGAGCACGGAAAGACGATGACGGAAAAAGAGATCGTGGATTA<br>CGTCGCCAGTCAAGTAACAACCGCGAAAAAGTTGCGCGGAGGAGTTGTGTTTGTGGACGAAGTA<br>CCGAAAGGTCTTACCGGAAAACTCGACGCAAGAAAAATCAGAGAGATCCTCATAAAGGCCAAGA<br>AGGGCGGAAAGATCGCCGTG

RLuc	ATGACTTCGAAAGTTTATGATCCAGAACAAAGGAAACGGATGATAACTGGTCCGCAGTGGTGGG<br>CCAGATGTAAACAAATGAATGTTCTTGATTCATTTATTAATTATTATGATTCAGAAAAACATGCAG<br>AAAATGCTGTTATTTTTTTACATGGTAACGCGGCCTCTTCTTATTTATGGCGACATGTTGTGCCACA<br>TATTGAGCCAGTAGCGCGGTGTATTATACCAGACCTTATTGGTATGGGCAAATCAGGCAAATCTG<br>GTAATGGTTCTTATAGGTTACTTGATCATTACAAATATCTTACTGCATGGTTTGAACTTCTTAATTT<br>ACCAAAGAAGATCATTTTTGTCGGCCATGATTGGGGTGCTTGTTTGGCATTTCATTATAGCTATGA<br>GCATCAAGATAAGATCAAAGCAATAGTTCACGCTGAAAGTGTAGTAGATGTGATTGAATCATGG<br>GATGAATGGCCTGATATTGAAGAAGATATTGCGTTGATCAAATCTGAAGAAGGAGAAAAAATGG<br>TTTTGGAGAATAACTTCTTCGTGGAAACCATGTTGCCATCAAAAATCATGAGAAAGTTAGAACCA<br>GAAGAATTTGCAGCATATCTTGAACCATTCAAAGAGAAAGGTGAAGTTCGTCGTCCAACATTATC<br>ATGGCCTCGTGAAATCCCGTTAGTAAAAGGTGGTAAACCTGACGTTGTACAAATTGTTAGGAATT<br>ATAATGCTTATCTACGTGCAAGTGATGATTTACCAAAAATGTTTATTGAATCGGACCCAGGATTCT<br>TTTCCAATGCTATTGTTGAAGGTGCCAAGAAGTTTCCTAATACTGAATTTGTCAAAGTAAAAGGTC<br>TTCATTTTTCGCAAGAAGATGCACCTGATGAAATGGGAAAATATATCAAATCGTTCGTTGAGCGA<br>GTTCTCAAAAATGAACAA
NanoLuc	ATGGTCTTCACACTCGAAGATTTCGTTGGGGACTGGCGACAGACAGCCGGCTACAACCTGGACCA<br>AGTCCTTGAACAGGGAGGTGTGTCCAGTTTGTTTCAGAATCTCGGGGTGTCCGTAACTCCGATCC<br>AAAGGATTGTCCTGAGCGGTGAAAATGGGCTGAAGATCGACATCCATGTCATCATCCCGTATGAA<br>GGTCTGAGCGGCGACCAAATGGGCCAGATCGAAAAAATTTTTAAGGTGGTGTACCCTGTGGATG<br>ATCATCACTTTAAGGTGATCCTGCACTATGGCACACTGGTAATCGACGGGGTTACGCCGAACATG<br>ATCGACTATTTCGGACGGCCGTATGAAGGCATCGCCGTGTTCGACGGCAAAAAGATCACTGTAAC<br>AGGGACCCTGTGGAACGGCAACAAAATTATCGACGAGCGCCTGATCAACCCCGACGGCTCCCTG<br>CTGTTCCGAGTAACCATCAACGGAGTGACCGGCTGGCGGCTGTGCGAACGCATTCTGGCG
LgBiT	ATGGTCTTCACACTCGAAGATTTCGTTGGGGACTGGGAACAGACAGCCGCGTACAACCTGGACCA<br>AGTCCTTGAACAGGGAGGTGTGTCCAGTTTGCTGCAGAATCTCGCGGTGTCCGTAACTCCGATCC<br>AAAGGATTGTCCGCAGCGGTGAAAATGCGCTGAAGATCGACATCCATGTCATCATCCCGTATGAA<br>GGTCTGAGCGCGGACCAAATGGCACAGATCGAAGAAGTGTTTAAGGTGGTGTACCCTGTGGATG<br>ATCATCACTTTAAGGTGATCCTGCCGTATGGCACACTGGTAATCGACGGGGTTACGCCGAACATG<br>CTGAACTATTTCGGACGGCCGTATGAAGGCATCGCCGTGTTCGACGGCAAAAAGATCACTGTAAC<br>AGGGACCCTGTGGAACGGCAACAAAATTATCGACGAGCGCCTGATCACCCCCGACGGCTCCATG<br>CTGTTCCGAGTAACCATCAACAGC

Nterm-His-eGFP	ATGGGCCATCACCATCATCACCATCACCACGGAGGTGGCACTAGTATGGAGCTTTTCACTGGCGT<br>TGTTCCCATCCTGGTCGAGCTGGACGGCGACGTAAACGGCCACAAGTTCAGCGTGTCCGGCGAG<br>GGCGAGGGCGATGCCACCTACGGCAAGCTGACCCTGAAGTTCATCTGCACCACCGGCAAGCTGC<br>CCGTGCCCTGGCCCACCCTCGTGACCACCCTGACCTACGGCGTGCAGTGCTTCAGCCGCTACCCCG<br>ACCACATGAAGCAGCACGACTTCTTCAAGTCCGCCATGCCCGAAGGCTACGTCCAGGAGCGCACC<br>ATCTTCTTCAAGGACGACGGCAACTACAAGACCCGCGCCGAGGTGAAGTTCGAGGGCGACACCC<br>TGGTGAACCGCATCGAGCTGAAGGGCATCGACTTCAAGGAGGACGGCAACATCCTGGGGCACAA<br>GCTGGAGTACAACTACAACAGCCACAACGTCTATATCATGGCCGACAAGCAGAAGAACGGCATC<br>AAGGTGAACTTCAAGATCCGCCACAACATCGAGGACGGCAGCGTGCAGCTCGCCGACCACTACC<br>AGCAGAACACCCCCATCGGCGACGGCCCCGTGCTGCTGCCCGACAACCACTACCTGAGCACCCAG<br>TCCGCCCTGAGCAAAGACCCCAACGAGAAGCGCGATCACATGGTCCTGCTGGAGTTCGTGACCG<br>CCGCCGGGATC
Cterm-His-eGFP	ATGGAGCTTTTCACTGGCGTTGTTCCCATCCTGGTCGAGCTGGACGGCGACGTAAACGGCCACAA<br>GTTCAGCGTGTCCGGCGAGGGCGAGGGCGATGCCACCTACGGCAAGCTGACCCTGAAGTTCATC<br>TGCACCACCGGCAAGCTGCCCGTGCCCTGGCCCACCCTCGTGACCACCCTGACCTACGGCGTGCA<br>GTGCTTCAGCCGCTACCCCGACCACATGAAGCAGCACGACTTCTTCAAGTCCGCCATGCCCGAAG<br>GCTACGTCCAGGAGCGCACCATCTTCTTCAAGGACGACGGCAACTACAAGACCCGCGCCGAGGT<br>GAAGTTCGAGGGCGACACCCTGGTGAACCGCATCGAGCTGAAGGGCATCGACTTCAAGGAGGA<br>CGGCAACATCCTGGGGCACAAGCTGGAGTACAACTACAACAGCCACAACGTCTATATCATGGCCG<br>ACAAGCAGAAGAACGGCATCAAGGTGAACTTCAAGATCCGCCACAACATCGAGGACGGCAGCGT<br>GCAGCTCGCCGACCACTACCAGCAGAACACCCCCATCGGCGACGGCCCCGTGCTGCTGCCCGACA<br>ACCACTACCTGAGCACCCAGTCCGCCCTGAGCAAAGACCCCAACGAGAAGCGCGATCACATGGTC<br>CTGCTGGAGTTCGTGACCGCCGCCGGGATCACGCGTGGAGGTGGCCATCACCATCATCACCATCA<br>CCACGGATAA

Table S3 Cloned plasmid information

Name	Purpose
pTXTL-T7max-H3H	H3H TXTL expression
pTXTL-T7max-Luz	Luz TXTL expression
pTXTL-T7max-LuxA	LuxA TXTL expression
pTXTL-T7max-LuxB	LuxB TXTL expression
pTXTL-T7max-LuxC	LuxC TXTL expression
pTXTL-T7max-LuxD	LuxD TXTL expression
pTXTL-T7max-LuxE	LuxE TXTL expression
pTXTL-T7max-Fre	Fre TXTL expression
pTXTL-T7max-FLuc	FLuc TXTL expression
pTXTL-T7max-RLuc	RLuc TXTL expression
pTXTL-T7max-NanoLuc	NanoLuc TXTL expression
pTXTL-T7max-HisGFPLuz	N-terminal His-tag eGFP fused Luz TXTL expression
pTXTL-T7max-LuzGFPHis	C-terminal His-tag eGFP fused Luz TXTL expression
pTXTL-T7max-HisGFPHiBiT	N-terminal His-tag eGFP fused HiBiT TXTL expression
pTXTL-T7max-HiBiTGFPHis	C-terminal His-tag eGFP fused HiBiT TXTL expression
pLumi-H3H	H3H carrying Rosetta 2 extract preparation
pLumi-LgBiT	LgBiT carrying Rosetta 2 extract preparation
